# A potential candidate gene associated with the angles of the ear leaf and the second leaf above the ear leaf in maize

**DOI:** 10.1186/s12870-023-04553-9

**Published:** 2023-11-04

**Authors:** Tianhui Kuang, Can Hu, Ranjan Kumar Shaw, Yudong Zhang, Jun Fan, Yaqi Bi, Fuyan Jiang, Ruijia Guo, Xingming Fan

**Affiliations:** 1 Institute of Food Crops, Yunnan Academy of Agricultural Sciences, Kunming, China; 2 School of Agriculture, Yunnan University, Kunming, China

**Keywords:** Maize, Ear leaf angle, Angle of the second leaf above the ear leaf, GWAS, QTL

## Abstract

**Background:**

Leaf angle is a key trait for maize plant architecture that plays a significant role in its morphological development, and ultimately impacting maize grain yield. Although many studies have been conducted on the association and localization of genes regulating leaf angle in maize, most of the candidate genes identified are associated with the regulation of ligule-ear development and phytohormone pathways, and only a few candidate genes have been reported to enhance the mechanical strength of leaf midrib and vascular tissues.

**Results:**

To address this gap, we conducted a genome-wide association study (GWAS) using the leaf angle phenotype and genotyping-by-sequencing data generated from three recombinant inbred line (RIL) populations of maize. Through GWAS analysis, we identified 156 SNPs significantly associated with the leaf angle trait and detected a total of 68 candidate genes located within 10 kb upstream and downstream of these individual SNPs. Among these candidate genes, *Zm00001d045408*, located on chromosome 9 emerged as a key gene controlling the angles of both the ear leaf and the second leaf above the ear leaf. Notably, this new gene’s homolog in Arabidopsis promotes cell division and vascular tissue development. Further analysis revealed that a SNP transversion (G/T) at 7.536 kb downstream of the candidate gene *Zm00001d045408* may have caused a reduction in leaf angles of the ear and the second leaf above the ear leaf. Our analysis of the 10 kb region downstream of this candidate gene revealed a 4.337 kb solo long-terminal reverse transcription transposon (solo LTR), located 3.112 kb downstream of *Zm00001d045408*, with the SNP located 87 bp upstream of the solo LTR.

**Conclusions:**

In summary, we have identified a novel candidate gene, *Zm00001d045408* and a solo LTR that are associated with the angles of both the ear leaf and the second leaf above the ear leaf. The future research holds great potential in exploring the precise role of newly identified candidate gene in leaf angle regulation. Functional characterization of this gene can help in gaining deeper insights into the complex genetic pathways underlying maize plant architecture.

**Supplementary Information:**

The online version contains supplementary material available at 10.1186/s12870-023-04553-9.

## Introduction

 Maize leaf angle is an important factor that influences the architecture of maize plants, and selection of maize varieties with an “ideal plant architecture” can effectively improve the utilization of light energy and increase maize yield [1, 2]. While factors such as variety selection, environmental conditions and cultivation practices largely determine the yield of maize [2, 3], a key factor contributing to modern maize yield improvement is the increase in planting density, which primarily depends on the architecture of the variety [4–7]. Thus, selecting compact varieties suitable for reasonable plant density is one of the important target traits in maize breeding programs aiming at improving maize yield [8].

A smaller leaf angle is one of the criteria for selecting the ideal plant architecture in maize breeding, as the middle leaves serve as the primary functional leaves of maize, and a compact upper leaves or small leaf angle can enable the middle leaves to receive more light intensity and promote biomass accumulation [9]. Researches have shown that when the upper ear leaves exhibit an upward leaf angle of approximately 20 ° to 25 °, and the lower ear leaves have a gentle angle of around 40 °, maize plants not only improve the light permeability in the maize field, enhance water and nutrients absorption through the roots, and slow down leaf senescence, but also improve photosynthetic efficiency in the central leaves, accelerate grain filling rate, and thus facilitate the accumulation of dry matter in maize [2, 10, 11].

Numerous research studies have been conducted to detect candidate genes that control leaf angles using methods such as GWAS, linkage analysis and other approaches. In one study, Tian et al [12] performed linkage analysis using a nested association mapping (NAM) population in maize and identified two key genes *Lg1* (Liguleless 1) and *Lg2* (Liguleless 2), located near the most significant QTLs on chromosomes 2 and 3. These two genes are known to regulate leaf angle. Lu et al [13] conducted GWAS on maize leaf angles and leaf orientation traits using 80 maize backbone inbred lines and detected 22 SNPs significantly associated with leaf angles, along with five candidate genes linked to leaf angle. Recently, Duan et al [14] identified four loci associated with leaf angles through GWAS involving 492 maize inbred lines. Similarly, Zhou et al [15] identified 18 candidate genes associated with leaf angle by GWAS on 573 maize F_1_ hybrid lines. All the candidate genes discovered in these studies are primarily related to regulatory mechanisms and can be broadly divided into four main groups: 1) those related to the regulation of ligule-ear development [16–23], 2) regulation of leaf polarity axis establishment [24–26], 3) regulation of the phytohormone pathway or the regulation of cell growth, differentiation and tissue development [27–34], and 4) regulation of mechanical tissue formation and vascular tissue development in the midvein region of the leaf [35].

Several candidate genes have been reported to be associated with leaf angle, with most of them being related to phytohormone metabolism. For instances, the gene *ZmDWF1* has been found to regulate maize leaf angle by affecting the expression of genes involved in phytohormone metabolism [36]. Additionally, two genes *ZmRPN10* [37] and *ZmbHLH112* [38] have been found to function similarly to the gene *ZmDWF1*. However, to date, only a few, if any, genes have been found to be linked to the enhancement of mechanical strength of maize leaves. In fact, researchers have shown that the mechanical strength of a leaf is an important trait that impact leaf angle [35]. This indicates a research gap that requires further investigation. Therefore, the present study was carried out with the following objectives: 1) To investigate if any molecular markers are associated with the angles of leaves at various positions in maize plant by GWAS using a multi-parent population, and 2) to identify candidate genes that may control the mechanical strength of leaves, which in turn impacts the angles of the leaves at specific positions in maize.

## Results

### Distribution of leaf angles of the ear leaf and the second leaf above the ear leaf for 465 maize RILs

**Fig. 1:**
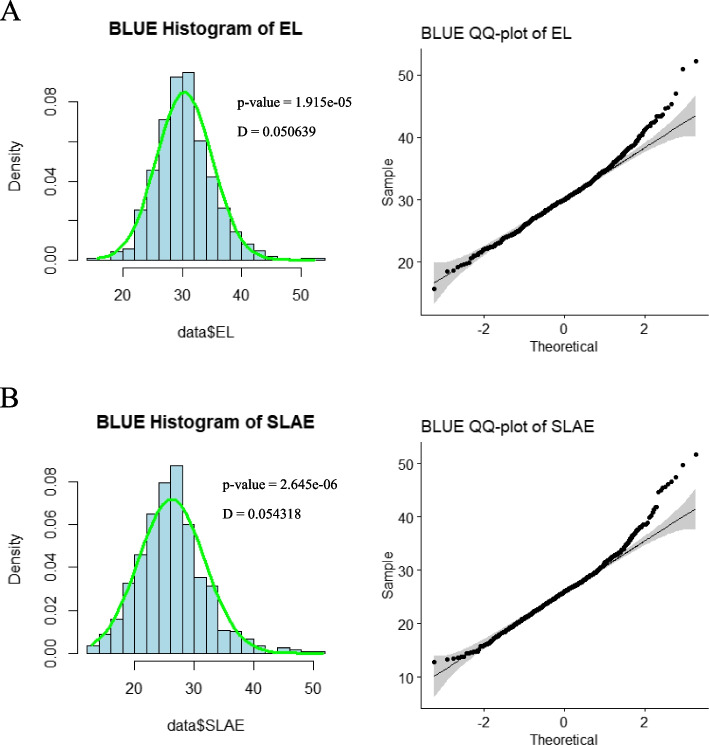
The normality test of BLUE value of the ear leaf and the second leaf above the ear leaf.  **A**  displays phenotype data distribution and normality test of the ear leaf. **B**  displays phenotype data distribution and normality test of the second leaf above the ear leaf. Histogram illustrates the phenotype data normality test, and the green line indicates the normal curve. QQ plot illustrate correlation between phenotype data and normal distribution. The black line indicates the 45 degree reference line.  Note: EL represents the ear leaf, SLAE represents the second leaf above the ear leaf, BLUE (Best Linear Unbiased estimate)

The data for leaf angles at five different positions were collected. A histogram with a normal distribution curve are presented in Fig. 1 for the leaf angles of the ear leaf and the second leaf above the ear leaf for BLUE (Best Linear Unbiased Estimates) of all 465 RILs. The figure demonstrated that the leaf angles of the ear leaf and the second leaf above the ear leaf for BLUE fit the normal distribution approximately with Kolmogorov-Smirnov normality test (Fig. 1). Similarly, for all three subpopulations, the majority of leaf angles also exhibited an approximately normal distribution (Fig. S1). These results support for further analysis.

### Genotyping-by-sequencing and SNP data profile

We employed the GBS (Genotyping-By-Sequencing) approach to sequence 465 RILs. After filtering, the average clean reads for each RIL was 3.78 Gb with a depth of 12.68X and a coverage of 12.12%. On average, the alignment rate of the samples were 98.56% and the coverage of at least four bases was 4.91%. The results indicated that the sequencing coverage for each sample was sufficient to adequately cover the reference genome, meeting the requirements for re-sequencing analysis. The clean reads were aligned with the maize B73 reference genome (ftp://ftp.ensemblgenomes.org/pub/release-40/plants/fasta/zea_mays/dna/Zea_mays.AGPv4.dna.toplevel.fa.gz) using BWA [39]. The alignment was performed with the following parameters: mem -t 4 -k 32 -M. Based on the alignment file, we identified and marked the duplicated SNPs without any deletion. For SNP detection and extraction, we followed the recommended process outlined in the following protocol: (https://gatk.broadinstitute.org/hc/en-us/articles/360036194592-Getting-started-with-GATK4).

### Population structure of 465 maize RILs

**Fig. 2:**
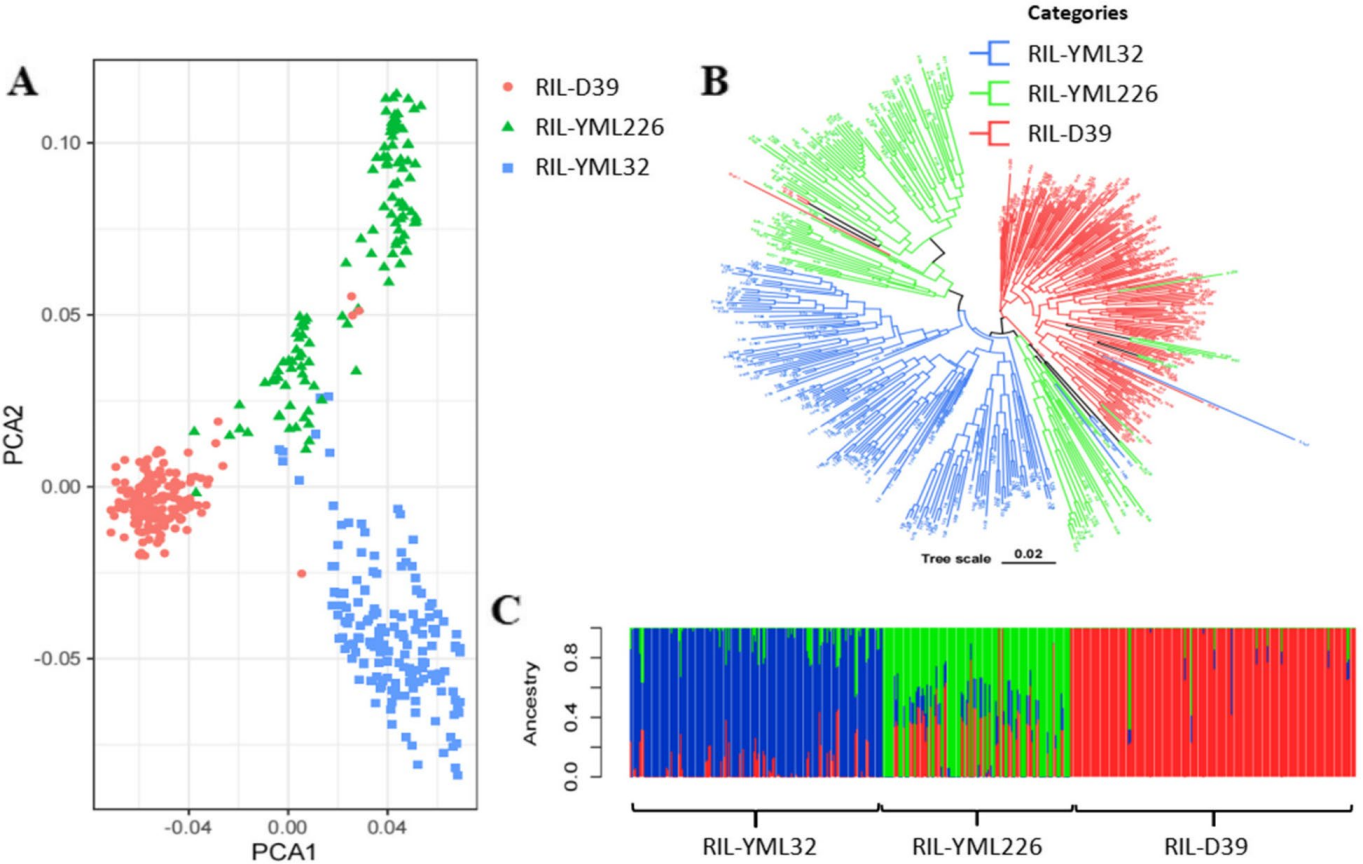
Population structure of 465 RILs. **A** The principal component analysis displays the clustering of RILs (Recombinant Inbred Lines), red dots represent RIL-D39, green triangles represent RIL-YML226, and blue squares represent RIL-YML32. **B** The evolutionary tree, with red branches representing RIL-D39, green branches representing RIL-YML226, and blue branches representing RIL-YML32. ** C** Ancestral component analysis, where red bars represent RIL-D39, green bars represent RIL-YML226, and blue bars represent RIL-YML32

The principal component analysis (PCA) (Fig. 2A) revealed clear and distinct classification of the 465 maize RILs from the three RIL populations into three main groups, which is in agreement with the “tri-heterotic group“ [40] theory. For instance, RIL-YML226 was classified as the non-Reid group, while RIL-YML32 and RIL-D39 were classified as the Suwan group. The evolutionary tree, constructed using MEGA (Fig. 2B), further supported the tri-heterotic group theory by grouping the 465 maize RILs into three major clusters. The figure indicated that RIL-YML32 and RIL-D39 populations were not strongly related to each other or had weak affinities between them, while there was some confusion between the evolutionary trees between the RIL-YML226 and RIL-YML32 populations. These results could be attributed to gene introgression during the breeding process of RIL development (Fig. 2C). Subsequent ancestral component analysis confirmed the findings of the PCA and the evolutionary tree, providing a clear, distinct and delineated population structure which was consistent when considering three subgroups (Fig. 2C).

### LD decay assessment

We used the raw SNP dataset for LD (Linkage Disequilibrium) decay analysis. We calculated the LD delay for each population, and the physical distance was about 10–20 kb when the rate of r^2^ decrease was leveled off (Fig. S2). Meanwhile, we took into account that the longest repeat element in the maize genome is 10 Kb, and therefore we chose 10 Kb as the criterion for screening candidate genes.

### Genome-wide association study of the angles of the ear leaf and of the second leaf above the ear leaf

**Fig. 3:**
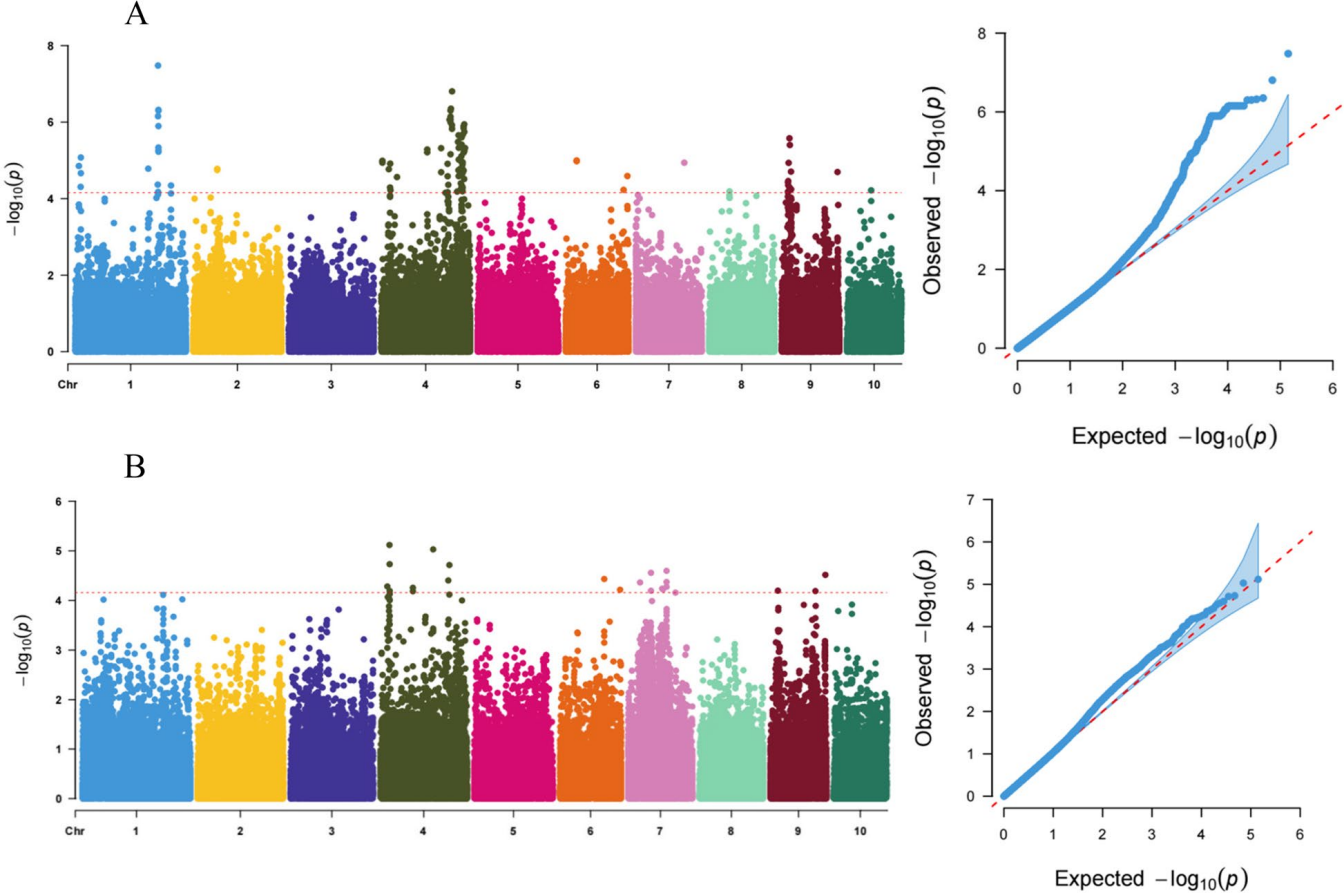
GWAS Results of the ear leaf and the second leaf above the ear leaf. **A** Manhattan plot (left) and QQ plot (right) for the ear leaf angle, and (**B**) Manhattan plot (left) and QQ plot (right) for angle of the second leaf above the ear leaf. The red dashed line in the Manhattan plot indicates the significance threshold, and the different colored dots represent the physical position of SNPs on the corresponding chromosomes and the y-axis represents the negative logarithmic values of significance with a base of 10. The red dashed line in the QQ plot indicates the expected significance value, and the blue dots indicate the actual significance value

Genome-wide association study was conducted based on the BLUE values of leaf trait data from the RILs. The linear mixed model (LMM) in GEMMA v0.98.5 was used for the analysis, combining 143,509 SNPs and BLUE values of the angles of the second leaf above the ear leaf and the ear leaf. SNPs with a *P*-value threshold < 6.960347e-05 were considered significantly associated with the trait (Fig. 3). GWAS analysis identified a total of 156 SNPs significantly associated with leaf angle traits (Table S1). Among them, 133 SNPs were significantly associated with the angle of the ear leaf and these SNPs were distributed on chromosomes 1, 2, 4, 6, 7, 8, 9 and 10 of maize (Fig. 3A, Table S1). Additionally, 23 SNPs were significantly associated with the angle of the second leaf above the ear leaf and were distributed on chromosomes 4, 6, 7 and 9 (Fig. 3B, Table S1).

**Fig. 4:**
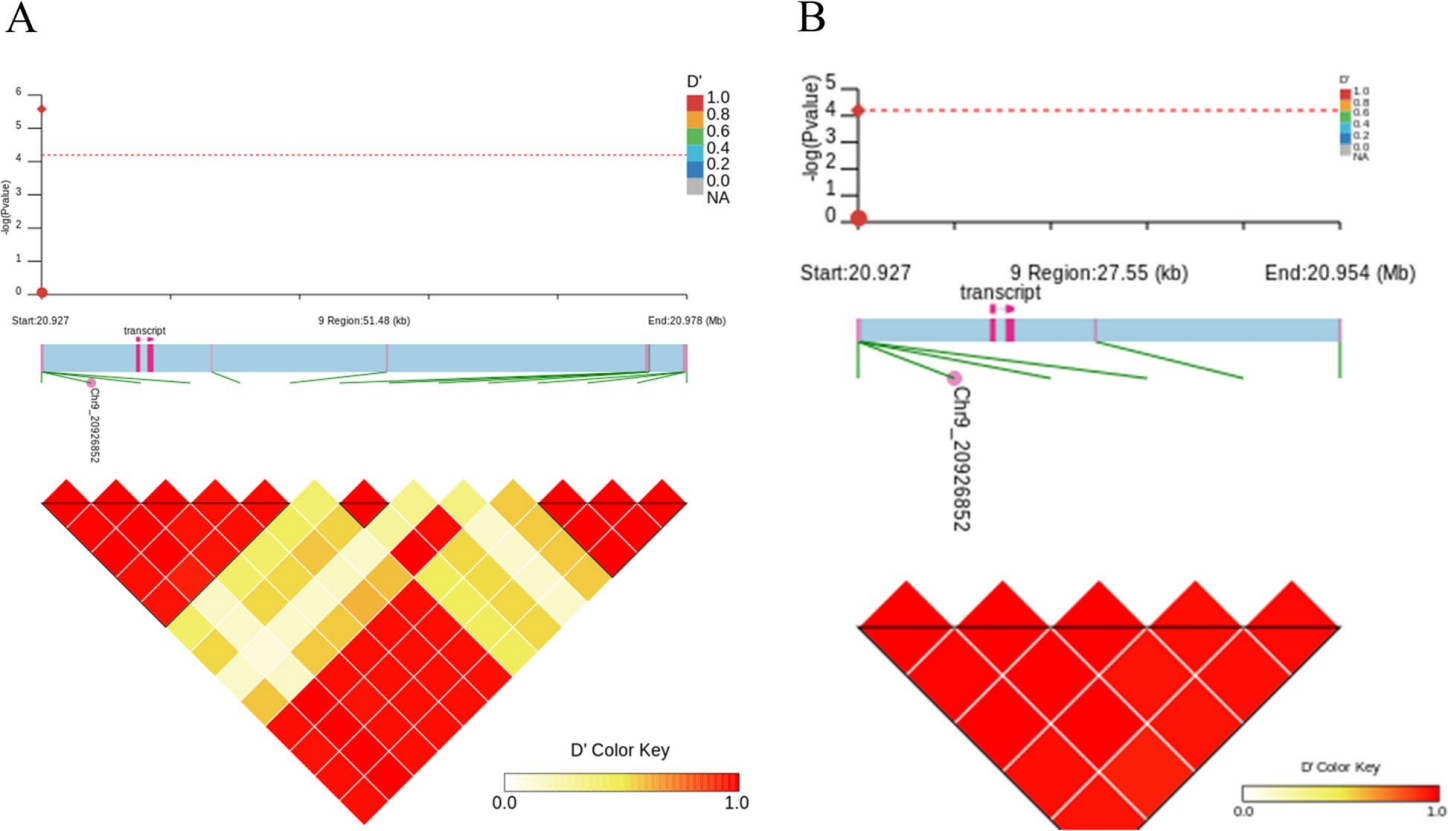
Linked SNPs of the most significant SNP.  **A** Represents the most significant SNPs associated with the ear leaf angle and the closely linked SNPs in their vicinity, **B** Represents the most significant SNPs associated with the angle of the second leaf above the ear leaf and the closely linked SNPs in their vicinity. Red diamond dots indicate the most significant SNPs, red dots indicate the most strongly linked SNP loci, red dashed lines indicate the significance threshold, pink squares indicate candidate gene transcripts and pink triangles indicate the upstream side of the transcript. The lower inverted triangle composed of red squares indicates the linkage strength of each SNP

A total of 14 candidate genes were identified from the 23 SNPs significantly associated with the angle of the second leaf above the ear leaf and they are located on chromosomes 4, 6, 7 and 9 (Tables S2, S3). Furthermore, from the 133 SNPs significantly associated with the angle of ear leaf, a total of 55 candidate genes were uncovered and they are located on chromosomes 1, 4, 6 and 9 (Table S2, S3). Notably, the gene *Zm00001d045408*, located on chromosome 9, was found to be co-localized for the angles of the ear leaf and the second leaf above the ear leaf. The most significant SNPs for these two leaf angles are linked with several other SNPs (Fig. 4).

### Effect of significant SNPs on phenotype

**Fig. 5:**
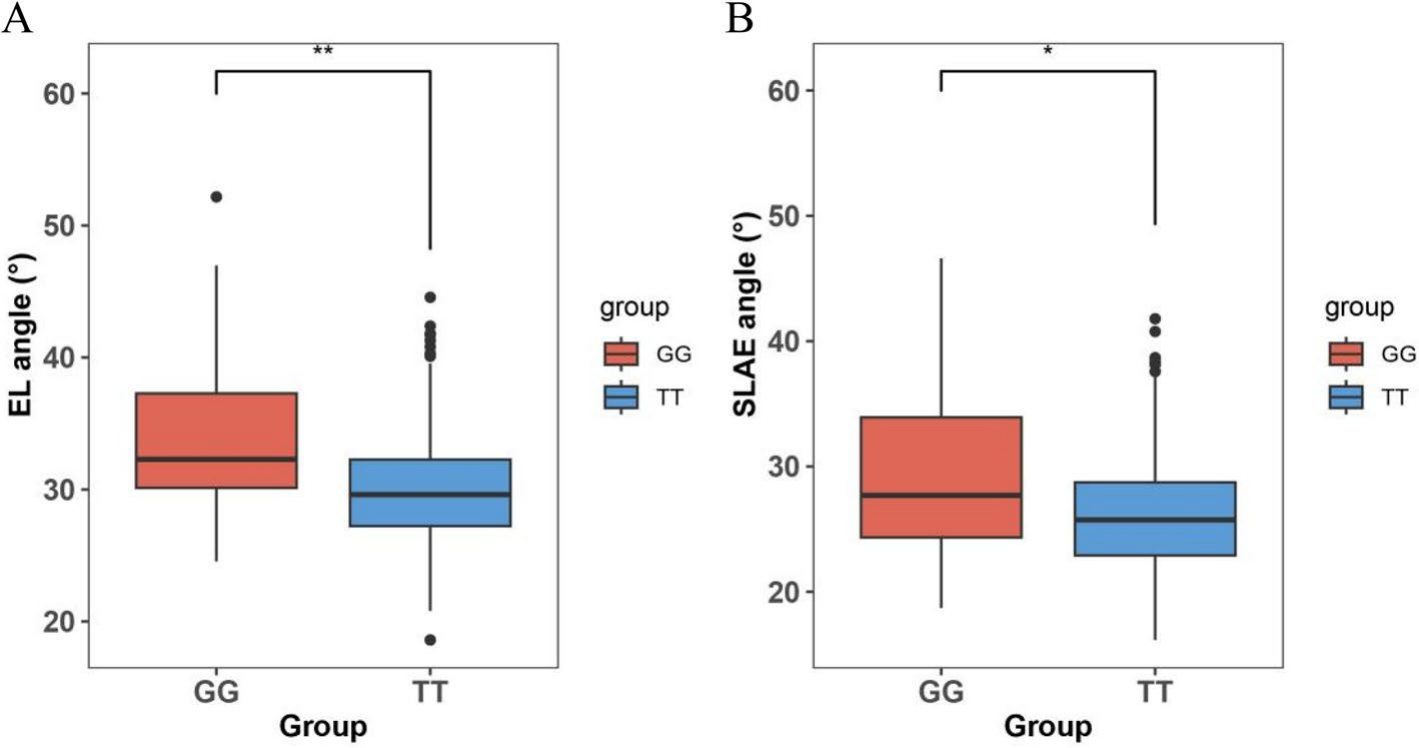
Effect of the type of the most significant SNP on the phenotype.  **A** Illustrates the difference in the corresponding phenotype between the two groups of RILs and the changes observed when the most significant SNP associated with the ear leaf angle is reversed from GG to TT. **B** Displays the difference in corresponding phenotype between the two groups of RILs and the changes observed when the most significant SNPs associated with the angle of the second leaf above the ear leaf are reversed from GG to TT.  Note: ** represents highly significant *p*  < 0.01, * represents significant *p*  < 0.05. EL represent the ear leaf, SLAE represent the second leaf above the ear leaf

**Fig. 6:**
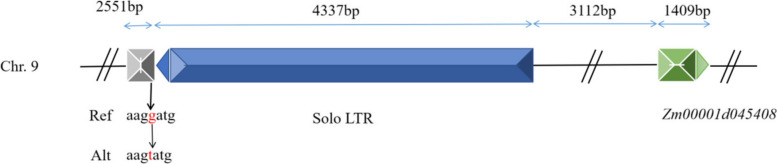
The relative positions of the most significant SNPs, solo LTRs and the genes.  Green squares indicate genes and green triangles indicate gene orientation; blue squares indicate long terminal repeat reverse transcription transposons (solo LTR) with independent insertions and blue triangles indicate transposon orientation. The gray square indicates the 2.551-kb sequence upstream of the transposon. Black arrows indicate the most significant SNP positions and the type of inversions

The SNP located at physical position 20926852 on chromosome 9 was identified as the significant SNP associated with the candidate gene *Zm00001d045408*. This SNP is positioned 7.536 kb downstream of the candidate gene and has a guanine (G) base on the reference genome. Among 422 out of the 465 RIL, the G base was reversed to thymine (T) (Fig. 5, Table S4). The base transversion (G/T) at position 20,926,852 on chromosome 9 resulted in a significant difference in leaf angles between the GG base-type group and the TT base-type group in the 465 RILs. The GG base-type group exhibited a greater angle of the ear leaf compared to the TT base-type group (t-test, *P*-value = 0.0045) (Fig. 5A). Similarly, the angle of the second leaf above the ear leaf significantly decreased after base transversion from G to T (t-test, *P*-value = 0.026) (Fig. 5B). Furthermore, upon annotating the 10-kb sequence downstream of the candidate gene, we, surprisingly, identified a solo long terminal reverse transcription transposon (solo LTR) with a length of 4.337 kb, located at 3.112 kb downstream of the candidate gene. Notably, the significant SNP was found to be located 87 bp upstream of the solo LTR (Fig. 6).

### GO enrichment analysis of candidate genes

We performed GO (Gene Ontology) enrichment analysis on 68 candidate genes. In the enrichment analysis results, we found that *Zm00001d032527*, *Zm00001d027636*, *Zm00001d051680*, *Zm00001d051682*, *Zm00001d052303*, *Zm00001d053055*, *Zm00001d053135*, *Zm00001d045233* and *Zm00001d052673* were enriched to GO:0003824 (GO term: catalytic activity). And *Zm00001d052436*, *Zm00001d039154*, *Zm00001d045268* and *Zm00001d045269* were enriched to GO:0043229 (GO term: intracellular organelle). The candidate gene *Zm00001d045408* was enriched to GO:0031224 (GO term: intrinsic component of membrane) (Table S5).

## Discussion

Major hybrid heterotic groups of maize include Reid, Lancaster (Non-Reid), Iodent, Lvda red cob, Tangsipingtou, Self 330, Suwan, Salisbury White, Southern Cross, Dent and Flint [41–45]. The four populations we selected for this study were Ye107, YML32, YML226, and D39. The Ye107 belong to Reid heterotic group;the YML32 and D39 fall in Suwan heterotic group; and the YML22 belong to Non-Reid heterosis group. The research on improvement of maize plant architecture has always been a hot subject in the field of maize breeding. In recent years, there have been increasing reports of genetic studies on maize leaf angle using GWAS [12, 46, 47]. At the same time some candidate genes were cloned and validated, and their molecular mechanisms for regulating leaf angle were gradually revealed from teosinte, temperate germplasm or tropical germplasm [48]. Some of the candidate genes localized in this study are quite consistent with previous studies. For examples, two genes on chromosome 1, *Zm00001d027636* (9,637,254–9,638,102 kb) and *Zm00001d027637* (9,638,241–9,644,168 kb) for the ear leaf angle are located in the region related *qLA-1* (2,037,772–12,397,449 kb) reported by the previous authors [49] and also located in the QTL hotspot region (8.517–16.560 Mb) [48]; Three genes *Zm00001d027819* (14,448,014–14,448,412 kb), *Zm00001d027844* (15,012,548–15,013,896 kb) and *Zm00001d027845* (15,013,950–15,018,699 kb) on chromosome 1 from this study are located in the previously reported leaf angle related regions of *qSTLAJ1-14* (12,397,449–29,259,990 kb) [50] and *qSecLA1b* (12,397,449–29,259,990 kb) [51] and also located within the QTL hotspot region (8.517–16.560 Mb) [48]. In addition, the genes *Zm00001d045408* (20,934,388–20,935,797 kb) and *Zm00001d045516* (25,240,624–25,249,861 kb) on chromosome 9 are in close physical position to the previously reported *qLAA9* (30,659,110–65,037,759 kb), which related to the angle of first leaf above the ear [52]. The genes *Zm00001d047373* (128,051,621–128,058,240 kb) and *Zm00001d047374* (128,061,736–128,062,497 kb) on chromosome 9 are also closely to the physical position of *qLAB9* (114,987,100–122,565,708 kb) reported in previous studies, which is a major QTL related to the angle of the first leaf below ear [52].

We have successfully identified a co-localized gene for the angles of ear leaf and the second leaf above the ear leaf, *Zm00001d045408*. Authors believe it is a novel potential candidate gene related to leaf angle in maize. This gene encodes a member of the S-adenosyl-l-methionine-dependent methyltransferase superfamily protein in maize and is believed to control the angles of ear leaf and the second leaf above the ear leaf. This candidate gene is located on chromosome 9 in maize and is a homolog of *AT5G40830*, a gene that encodes a similar protein in Arabidopsis. Additionally, we have identified a solo LTR spanning 4.337 kb, located at 3.112 kb downstream of this candidate gene, and the significant SNP is located 87 bp upstream of the LTR (Fig. 6).

Previous studies had demonstrated that the overexpression of the *AT5G40830* gene in Arabidopsis results in elevated levels of cytokinins (CKs) and gibberellins (GAs), leading to an increase in the number of xylem and bast cells in Arabidopsis inflorescence stems, without altering the cell volume. This ultimately leads to the elongation of Arabidopsis inflorescence stems and an increase in plant biomass [53, 54]. Moreover, the overexpression of the *AT5G40830* gene has been proposed to accelerate methanol production and release in plants. Exogenous methanol has been shown to stimulate radial growth and stem elongation of inflorescence stems and promotes vascular tissue development [55, 56]. Studies focusing on the regulatory mechanism of maize leaf angle have suggested that maize can reduce leaf angle by increasing the number of tissue cells in the auricular region of the ligule and enhancing the mechanical strength of the ligule region [57]. Furthermore, it has been observed that increasing the mechanical strength of maize midrib and other leaf veins leads to a decrease leaf angle in maize [35].

Therefore, we hypothesize that this candidate gene, *Zm00001d045408*, a homolog of *AT5G40830*, may have a similar function in maize like its Arabidopsis counterpart. We speculate that the expression of this candidate gene in maize could enhance the number of xylem and bast cells, promoting the development of maize vascular tissue. Additionally, based on previous studies on the regulatory mechanism of maize leaf angle, we hypothesize that the expression of this candidate gene may also enhance the mechanical strength of maize leaf tissue, leading to a reduction in the maize leaf angle.

The plant genome harbors a considerable number of intergenic regions, which constitute approximately 85% of the maize genome [58, 59]. Initially, intergenic regions were considered as “junk DNA” due to the lack of direct functional characterization [60]. Despite significant advancements in sequencing technology and analytical tools, the functional studies of intergenic regions still face significant challenges [61]. However, recent progress has been made in understanding the functions of intergenic regions. For instance, an intergenic region of 5 kb located approximately 5 kb upstream of the rice *qSW21/GW5* gene has been shown to influence the width and weight of rice seeds [62]. Similarly, in maize, a tandem repeat sequence located 100 kb upstream of the *BOOSTER853* gene regulates anthocyanin biosynthesis by modulating the expression of this gene [63, 64]. Additionally, retrotransposon, such as one located 2 kb upstream of *ZmRap11.70*, affects the epigenetic modification of *ZmRAP7.2*, which in turn regulates the expression of *ZmRAP7.12*, leading to early flowering time [65, 66]. The *TEOSINTE BRANCHED1* (*TB1*) gene, a key gene in maize domestication, controls the number of tillers, with its expression being controlled by two transposons located 60 kb upstream of the gene [67]. Another intergenic region, *KERNEL ROW NUMBER4* (*KRN4*), approximately 3.1 kb long and located 60 kb downstream of the *UNBRANCHED3* (*UB3*) transcription factor gene, enhances the expression of *UB3* and leads to the flattening of the maize cob and changes in the number of rows. Intergenic regions positioned both upstream and downstream of genes can impact gene expression, regardless of their proximity to the gene [68], ultimately regulating quantitative traits. In this study, we identified an independently inserted long terminal reverse transcription transposon (solo LTR) located 3.112 kb downstream of the candidate gene, with a significant SNP located 87 bp upstream of this LTR for association analysis (Fig. 6). In apple, this independently inserted long terminal retrotransposon has shown promoter activity [69]. Thus, we hypothesize that this retrotransposon may regulate the candidate gene *Zm00001d045408*. Our results suggest that intergenic DNA plays an important role in controlling leaf angles in maize.

Leaf angle plays a crucial role in shaping maize plant architecture, and a desirable maize plant architecture is characterized by a smaller leaf angle. Selecting compact maize varieties with smaller leaf angles significantly enhances maize plant tolerance to high density and resistance to lodging during planting, ultimately contributing to maize yield improvement. Therefore, the identification of new candidate genes involved in the regulation of leaf angle can contribute to the development of valuable genetic resources and innovative ideas for breeding and selecting compact maize varieties.

## Conclusion

In summary, we have identified a novel candidate gene, *Zm00001d045408* and a solo LTR that are associated with the angles of both the ear leaf and the second leaf above the ear leaf. Through annotation and homologous gene function analysis in Arabidopsis, we propose that the candidate gene *Zm00001d045408* could be responsible for controlling cell division and vascular tissue development in maize leaf. In addition, the solo LTR may play a significant role in regulating the expression of the candidate gene.

The future research hold great potential in exploring the precise role of newly identified candidate gene in leaf angle regulation. Functional characterization of these genes can help in gaining deeper insights into the complex genetic pathways underlying maize plant architecture. Understanding the genetic basis of leaf angle regulation can contribute to sustainable agriculture practices by facilitating the development of compact maize varieties with improved tolerance to high planting densities and increased resistance to lodging.

## Materials and methods

### Selection of parental lines and multi-parent population development

**Fig. 7:**
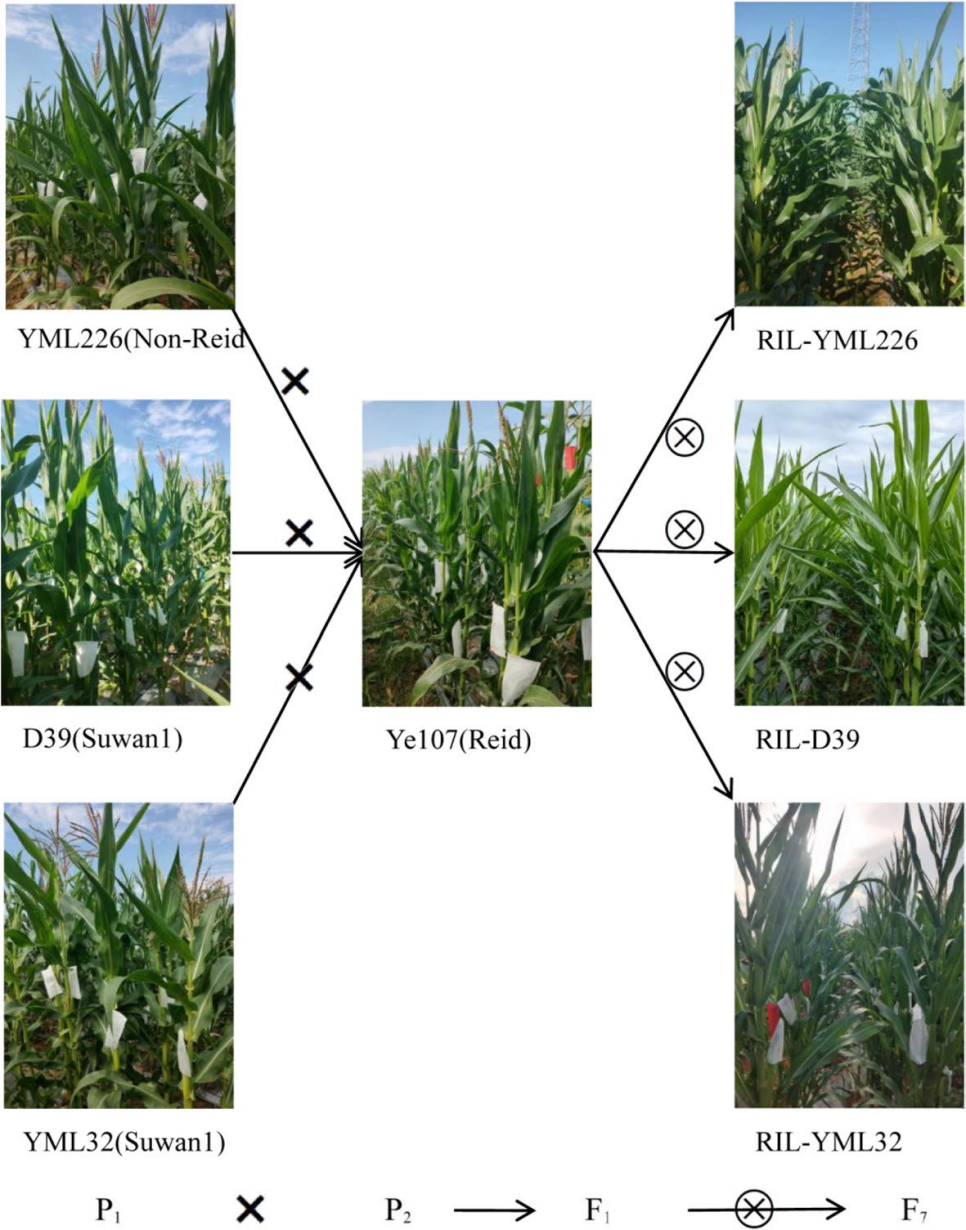
Schematic diagram of population construction. YML32, D39, YML226 and Ye107 are the four parents, with Ye107 being the common male parent. The F_1_ generation resulting from crossing these four parents was subjected to six consecutive rounds of self crossing to obtain the F_7_ generation

A multi-parent population was created by selecting the elite maize inbred line of Ye107 as the common male parent and three tropical backbone maize inbred lines, namely YML32, YML226, and D39, as female parents. The parental line’s names and lineage information for the respective RILs are presented in Table 1. Ye107 was selected as the common male parent since it is a representative of a maize inbred line with compact plant architecture and small leaf angles, cultivated across China. The three female parental lines were selected due to their widespread use in maize breeding programs, with several successful hybrids developed from them in southern China (Fig. 7). This multi-parent population consists of three F_7_ RIL sub-populations, viz. RIL-YML32, RIL-YML226, RIL-D39, totaling 465 F_7_ RILs. Initially, each RIL sub-population consisted of 200 individuals. However, a small proportion of these individuals was lost due to inbreeding depression and other stresses during the inbreeding process. Finally, the RIL-YML32 sub-population had 162 RILs, RIL-YML226 had 120 RILs, and RIL-D39 had 183 RILs. As a result, a total of 465 RILs remained and would be utilized for subsequent analysis.

**Table 1:** Names, pedigree, heterotic groups, and ecotype of the parental lines used in constructing the multi-parent populations

Parentage	Genealogy or pedigree	Heterotic Group	Ecotype
Ye107	Selected from commercial American hybrids	Reid	Temperate
YML32	Suwan 1(S)C9-S8-346-2 (Kei 8902)-3-4-4-6	Suwan	Tropical
YML226	(CML226/(CATETO DC1276/7619))F2-25-1-B-1-2-1-1-2(DH)	Non-Reid	Tropical
D39	Suwan1	Suwan	Tropical

### Phenotype evaluation of leaf angle trait

The phenotype data for leaf angles of the 465 F_7_ RILs were collected at three locations in Yunnan Province, namely Dehong (DH), Baoshan (BS) and Yanshan (YS), during the years 2018, 2019 and 2022, respectively. The experiment was conducted using a randomized block design (RBD) with three replications. Each RIL was planted in a single row with a spacing of 0.7 m and a row length of 3.2 m, containing 14 plants per row. The total plant density was approximately 62,112 plants/ha. Standard agronomic practices were followed throughout the trial.

At the R1 stage, three representative maize plants with relatively uniform leaf angles were selected, and the following leaf angles were measured for each RIL viz. the third leaf above the ear leaf, the second leaf above the ear leaf, the first leaf above the ear leaf, the ear leaf and the first leaf below the ear leaf. The phenotype data of leaf angles were analyzed using the psych v2.2.9 [70] package in R. The best linear unbiased estimation (BLUE) [71] of the lme4 v1.1-30 [72] package in R was used to obtain BLUE values for the angles of all the aforementioned leaves in Dehong, Baoshan, and Yanshan. The formula for calculating BLUE is as follows: LA ~ Cul + (1 | location) + (1 | year) + (1 | location: year: rep). Among them, Cul represents the variety as a fixed factor. In addition, (1 | location) represents location as a random factor, (1 | year) represents year as a random factor, and (1 | location: year: rep) represents the interaction between location year and variety as a random factor. These BLUE values were further utilized for genome-wide association study.

### Genotyping-by-Sequencing, SNP extraction and filtering

A Genotyping-by-sequencing (GBS) approach was performed for the 465 maize RILs. GBS libraries were constructed following the method described by Poland et al [73]. Briefly, genomic DNA was digested using PstI and MspI restriction enzymes (New England BioLabs, Ipswich, MA, United States), and barcoded adapters were ligated to the digested DNA fragments using T4 ligase (New England BioLabs). The ligated products from each plate were pooled and purified using the QIAquick PCR Purification Kit (QIAGEN, Valencia, CA, United States). PCR (Polymerase Chain Reaction) amplification was conducted using primers complementary to both adapters. The resulting PCR products were further purified with the QIAquick PCR Purification Kit and quantified using the Qubit dsDNA HS Assay Kit (Life Technologies). Size selection of fragments ranging from 200-bp to 300-bp was performed using an Egel system (Life Technologies), and the concentration of each library was estimated using a Qubit 2.0 fluorometer and the Qubit dsDNA HS Assay Kit (Life Technologies). The size-selected libraries were sequenced on an Ion Proton sequencer (Life Technologies, software version 5.10.1) using P1v3 chips after library preparation on an Ion Chef instrument (Ion PI HiQ Chef Kit). The Ion Torrent system generated sequence reads of variable lengths. After sequencing, raw data underwent filtering to remove adaptor and low-quality sequence. The clean reads obtained from the sequencing of RILs from YML32, YML226 and D39 populations were aligned to the maize B73 v4 genome to generate the bam file. SNP extraction was performed using the GATK optimal process (https://gatk.broadinstitute.org/hc/en-us/articles/360036194592-Getting-started-with-GATK4). Plink v 1.9 [74] was used to filter SNPs, with parameters set to --geno 0.2 and --maf 0.05(SNP deletion rate < 20 and minor allele frequency < 0.05) to filter out loci with deletion rates above 10% and loci with minimum allele frequencies less than 5%. The resulting filtered dataset was used for further analysis.

### Population structure assessment

The filtered SNP dataset was used for reconstructing the evolutionary tree. Prior to the reconstruction, chain imbalance filtering was performed using plink v1.9 with parameters set to --maf 0.05 --geno 0.2 --indep-pairwise 50 10 0.2(SNP deletion rate < 20; minor allele frequency < 0.05; a sliding window size of 50 SNPs, a minimum of 10 SNPs within the sliding window, and a maximum r^2^ threshold of 0.2). The filtered dataset was then used to reconstruct the evolutionary tree. To achieve this, we first converted the vcf file into phylip format using vcf2phylip.py v2.0 and then imported the converted file into MEGA v11.0 [75] to build the Neighbor-Joining (NJ) tree. The bootstrap set to 1000 and the rest of the parameters using the default values.

We also conducted a principal component analysis on the genotypic dataset, which was filtered with minor allele frequency. To accomplish this analysis, vcftools v1.7 [76] was first used to convert the vcf files into binary files, and then plink v1.9 was used to calculate the first ten principal components, and finally, the result was visualized using R.

The filtered data set was used to estimate population structure using Admixture v1.6 [77]. The number of subgroups K set from 1 to 30, with the remaining parameters set to default. The CV values in the log file were extracted, and the K value corresponding to the smallest CV error was considered as the optimal number of groups. The group results were visualized using ggplot2 v3.4.0. To calculate the kinship matrix between individuals, GEMMA v0.98.5 [78] was used with parameters set to -gk 2(calculates the standardized relatedness matrix).

### LD decay assessment

We used the raw SNP dataset for LD decay analysis. The software used for LD decay analysis was PopLDdecay v3.42 [79] with parameters set to default.

### Genome-wide association study

The raw SNP data were filtered using plink v1.9 with parameters set to --maf 0.05 --geno 0.2(SNP deletion rate < 20 and minor allele frequency < 0.05). The resulting filtered data was then utilized for genome-wide association study. The mixed-linear-model (MLM) in GEMMA (Genome-wide Efficient Mixed Model Association algorithm) was employed for GWAS. MLM is a highly efficient and accurate model compared to other mixed linear models, as it incorporates group structure and kinship as covariates [80]. The GWAS parameters were set to -lmm 1 [81]. The *P*-value threshold was calculated by Bonferroni Correction (significant level/SNPs used in GWAS). The results were visualized using CMplot v3.6.2 [82], where SNP loci with significant *P*-values were plotted within chromosomal regions. The horizontal axis of the plot represented the physical location of the locus, and the vertical axis represented the logarithmic value of the SNP’s *P*-value.

SNP loci that meet or exceed the threshold value were extracted using bedtools v1.7 [35]. Based on the B73 v4 reference genome sequence and annotation information, candidate genes associated with the leaf angle trait in maize were identified within a 10 kb upstream and downstream region where significantly associated SNPs were located. The 10 kb was used due to the facts that when r^2^ in LD decay chart, plateau was obtained at 10–20 kb and the longest repeat element in the maize genome is 10 Kb. The annotation and functional prediction of these candidate genes were based on information from the Uniprot protein database (https://www.uniprot.org/uniprot).

### Significant SNP locations and its types

We utilized LDBlockshow v1.40 [83] to identify SNP-linked blocks located within a 40 kb region upstream and downstream of the candidate gene. Meanwhile, vcftools v 0.1.16 [76] was used to extract the SNPs of all RILs at this locus, based on the chromosomal and physical location information of the most significant SNPs. Based on the SNP information of this location, the phenotype data of 465 RILs were divided into 2 groups and tested for differences.

### GO enrichment analysis of candidate genes

We performed GO enrichment analysis of the candidate genes. GO accessions and GO terms for the candidate genes were obtained. GO enrichment analysis was performed using the agriGO online website (http://systemsbiology.cau.edu.cn/agriGOv2/specises_analysis.php?&SpeciseID=20&latin=Zea_mays). The background is Maize v4 (Ensembl), the rest of the parameters are default.

### Supplementary Information

**Additional file 1:** **Figure S1.** The normality test of the BLUE values and the three RILs populations means. **Figure S2. **The LD decay of three populations. **Figure S3.** Effect of the most significant SNP type on phenotype in RIL-YML226. **Table S1.** Significant SNPs of the ear leaf and the second leaf above the ear leaf. **Table S2.** The positions and functions of genes scanned for significant SNPs of the ear leaf and the second leaf above the ear leaf. **Table S3.** Genes associated with Significant SNPs of the ear leaf and the second leaf above the ear leaf. **Table S4.** Number of most important SNP locus types in the three populations. **Table S5.** GO enrichment result of candidate genes.

## Data Availability

Te datasets presented in this study can be found in online repositories. Te names of the repository/repositories and accession number(s) can be found at: https://www.ncbi.nlm.nih.gov/, PRJNA983090.
