# Supplementary material for: A potential candidate gene associated with the angles of the ear leaf and the second leaf above the ear leaf in maize
Source: BMC Plant Biol. 2023 Nov 4;23:540. doi: 10.1186/s12870-023-04553-9 (PMC10625212; doi:10.1186/s12870-023-04553-9)
Supplement: Supplementary file 1 — Additional file 1: Figure S1. The normality test of the BLUE values and the three RILs populations means. Figure S2. The LD decay of three populations. Figure S3. Effect of the most significant SNP type on phenotype in RIL-YML226. Table S1. Significant SNPs of the ear leaf and the second leaf above the ear leaf. Table S2. The positions and functions of genes scanned for significant SNPs of the ear leaf and the second leaf above the ear leaf. Table S3. Genes associated with Significant SNPs of the ear leaf and the second leaf above the ear leaf. Table S4. Number of most important SNP locus types in the three populations. Table S5. GO enrichment result of candidate genes. [file 12870_2023_4553_MOESM1_ESM.docx]

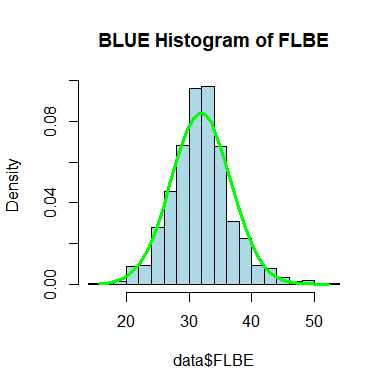

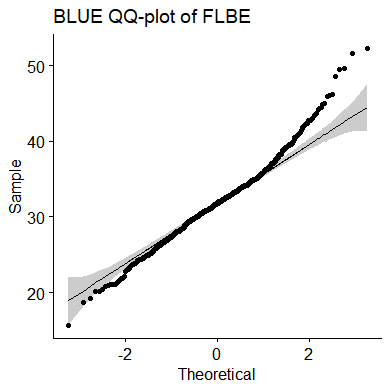


p-value = 1.432e-06

D = 0.055371


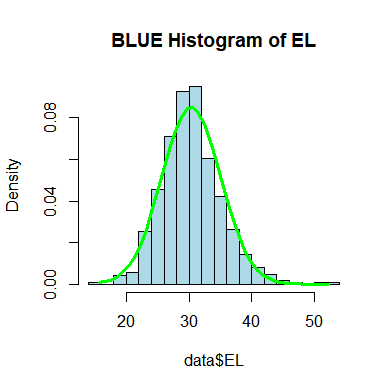

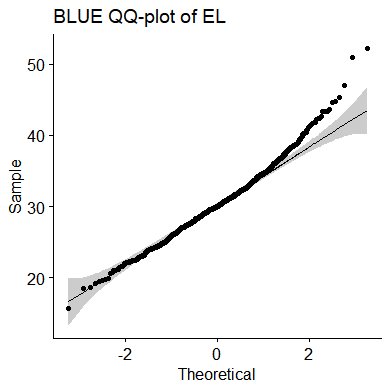


p-value = 1.915e-05

D = 0.050639


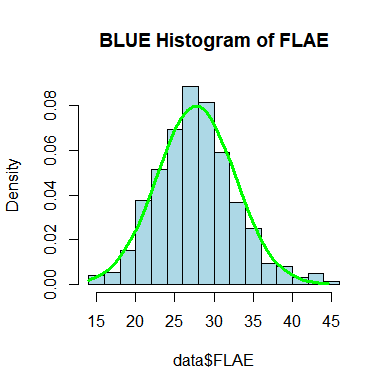

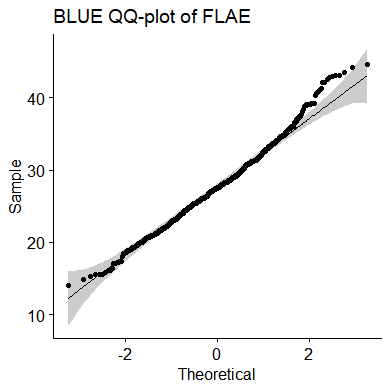


p-value = 0.0004703

D = 0.043914


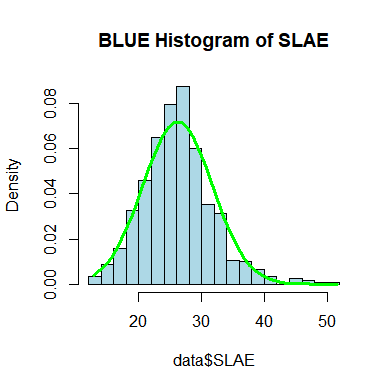

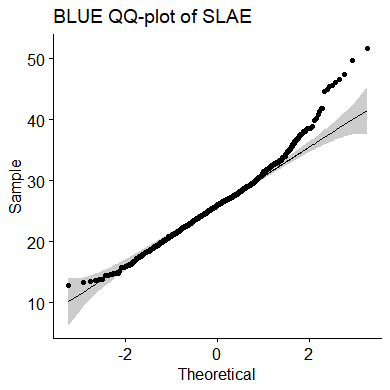


p-value = 2.645e-06

D = 0.054318


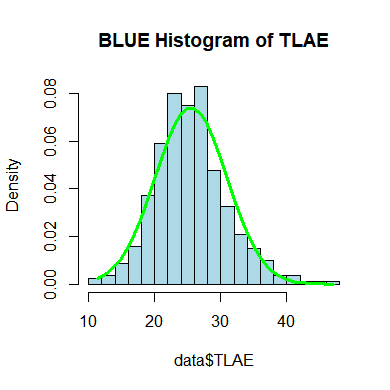

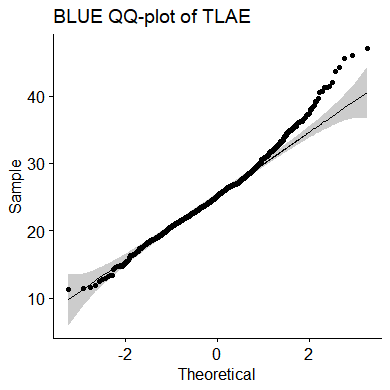


p-value = 1.8e-07

D = 0.058896


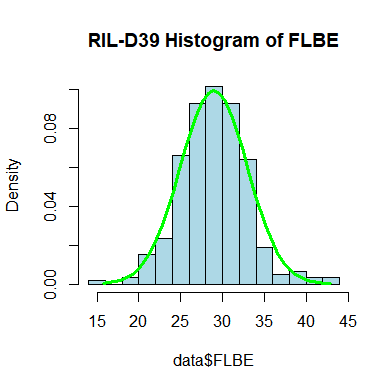

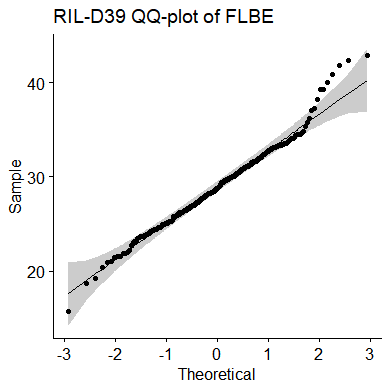


p-value = 0.3852

D = 0.037807


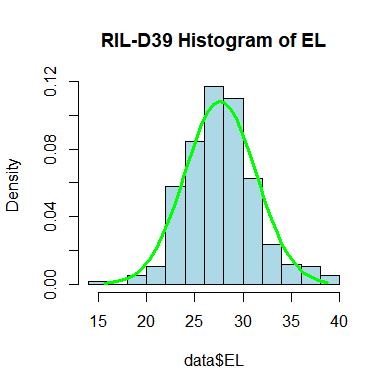

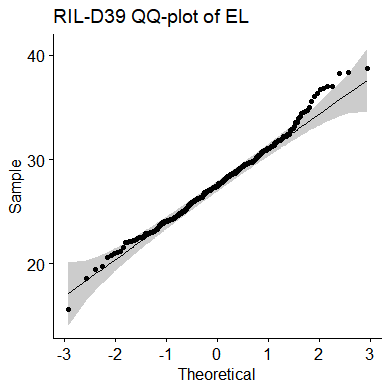


p-value = 0.2693

D = 0.040862


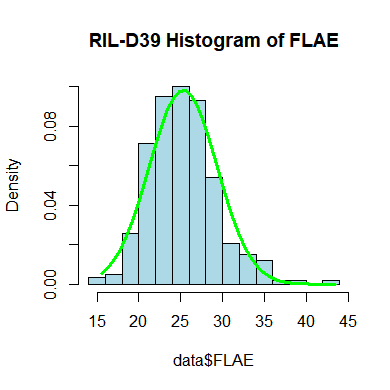

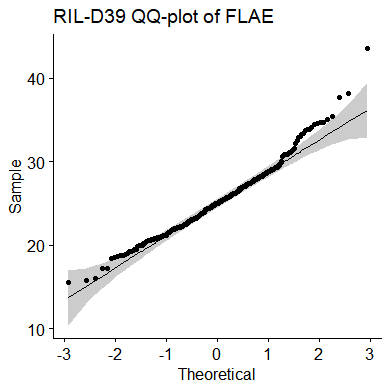


p-value = 0.07298

D = 0.049924


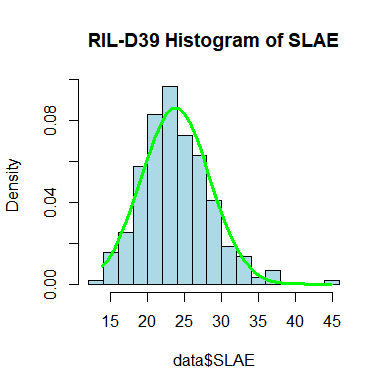

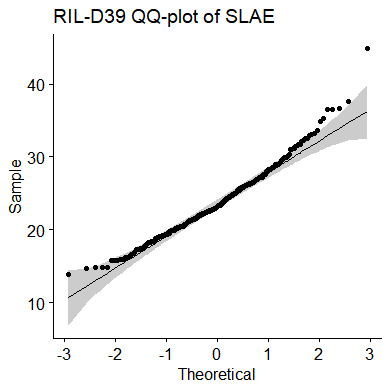


p-value = 0.01464

D = 0.059092


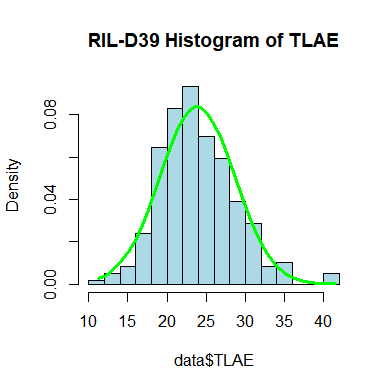

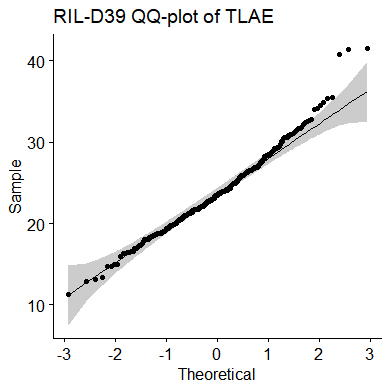


p-value = 0.01257

D = 0.059869


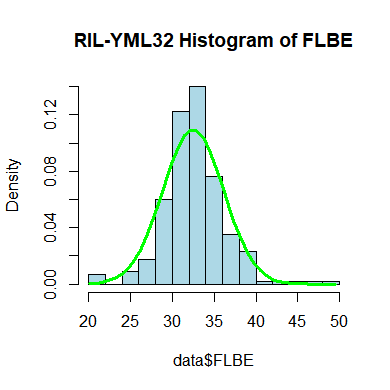

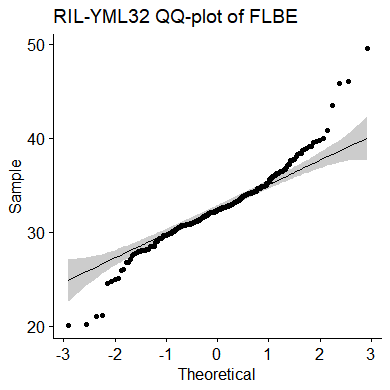


p-value = 0.0001723

D = 0.079835


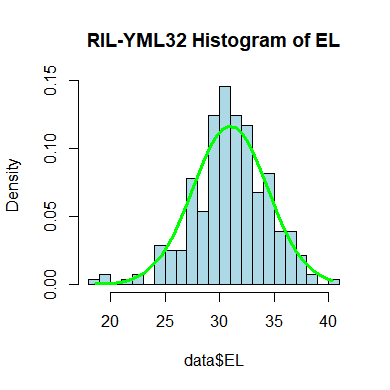

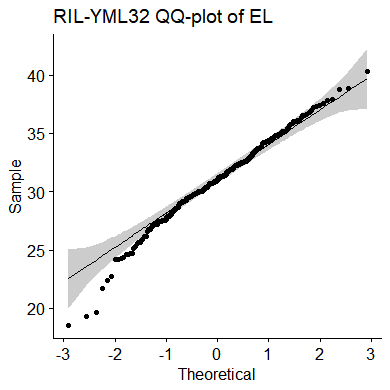


p-value = 0.05135

D = 0.053275


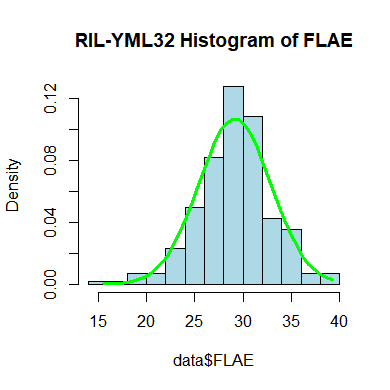

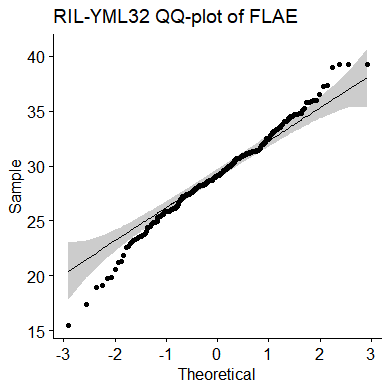


p-value = 0.01651

D = 0.059774


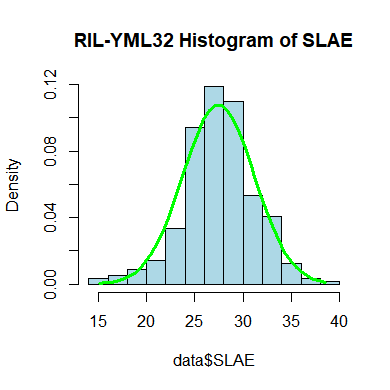

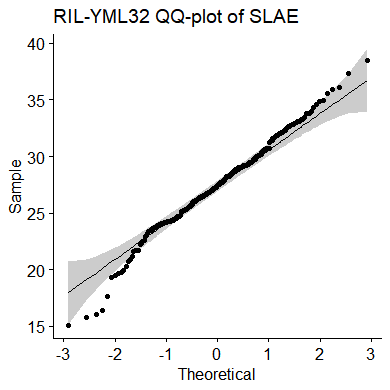


p-value = 0.0602

D = 0.05228


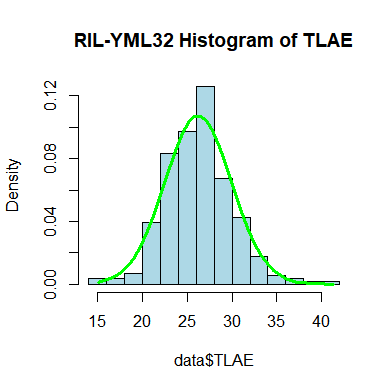

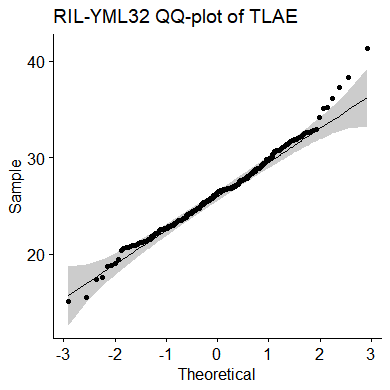


p-value = 0.02685

D = 0.057107


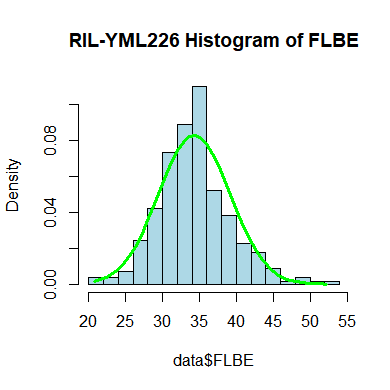

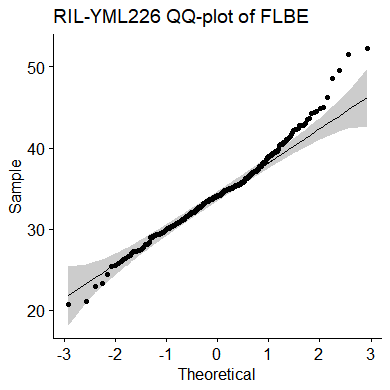


p-value = 0.0004873

D = 0.075176


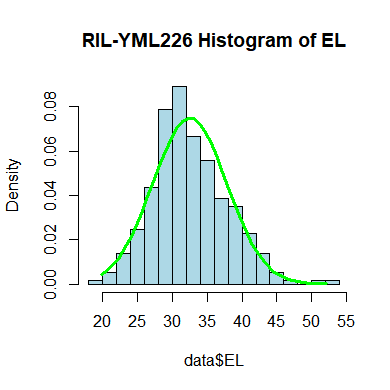

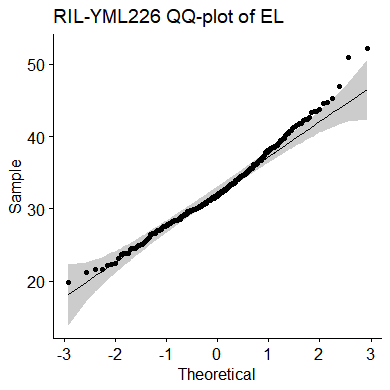


p-value = 0.01536

D = 0.059743


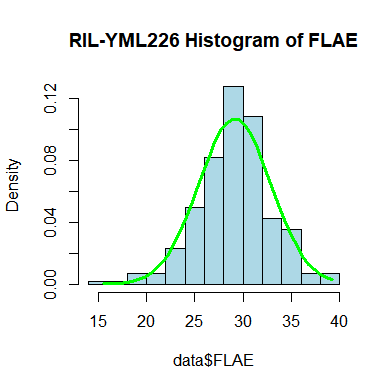

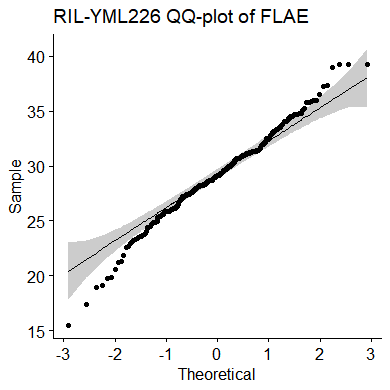


p-value = 0.01651

D = 0.059774


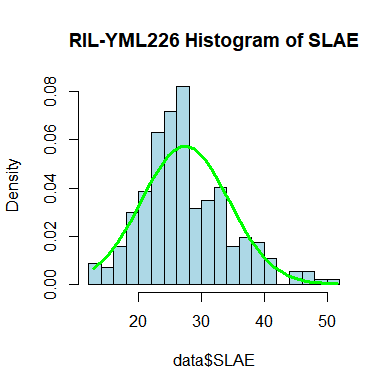

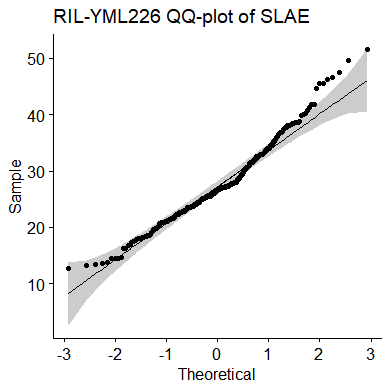


p-value = 2.953e-08

D = 0.1061


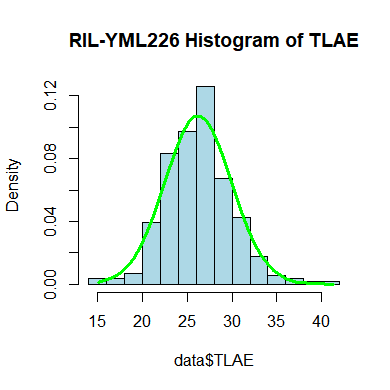

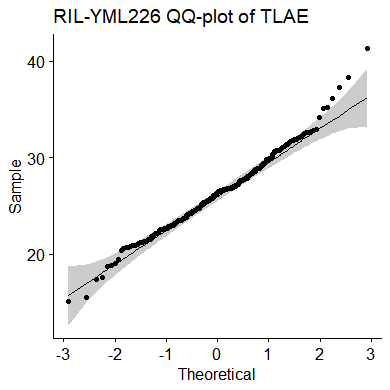


p-value = 0.02685

D = 0.057107

**Figure S1. The normality test of the BLUE values and the three RILs populations means.** FLBE represents the first leaf below the ear leaf, EL represents the ear leaf, FLAE represents the first leaf above the ear leaf, SLAE represents the second leaf above the ear leaf, and TLAE represents the third leaf above the ear leaf. Histogram illustrate phenotype data normality test (Lilliefors test), and the green line indicates the normal curve. QQ plot illustrate correlation between phenotype data and normal distribution. The black line indicates the 45 degree reference line.

Note: The smaller D indicates that the sample data is closer to a normal distribution. If the p-value is greater than the significance level α (0.05), it indicates that the sample data follows a normal distribution.


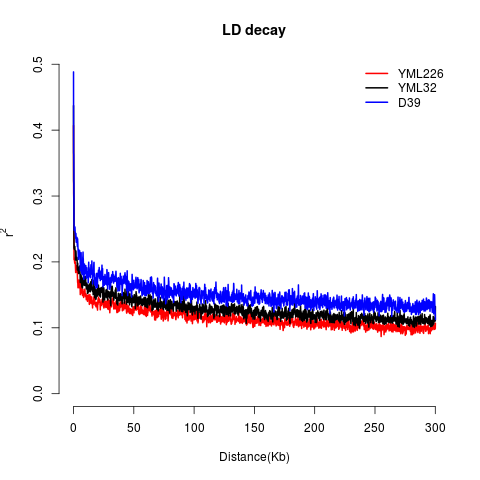


**RIL-YML226**

**RIL-YML32**

**RIL-D39**

**Figure S2.The LD decay of three populations.**

LD decay determined by squared correlations of allele frequencies (r^2^) against distance between polymorphic sites in RIL-YML226 (red), RIL-YML32 (black) and RIL-D39 (blue).

A B

**
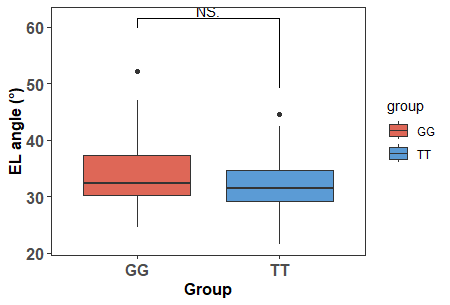

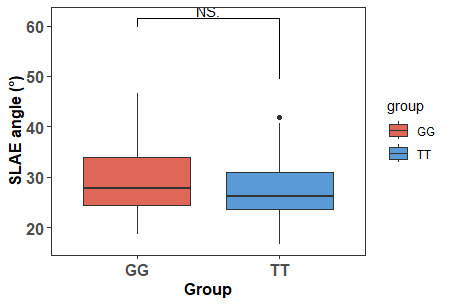
**

**Figure S3. Effect of the most significant SNP type on phenotype in RIL-YML226.**

1. Illustrates the difference in the corresponding phenotype between the two groups of RIL-YML226 and the changes observed when the most significant SNP associated with the ear leaf angle is reversed from GG to TT. (B) Displays the difference in corresponding phenotype between the two groups of RIL-YML226 and the changes observed when the most significant SNPs associated with the angle of the second leaf above the ear leaf are reversed from GG to TT.

Note: NS. represents no significance. EL represent the ear leaf, SLAE represent the second leaf above the ear leaf.

**Table S1: Significant SNPs of the ear leaf and the second leaf above the ear leaf.**

| Chromesome | SNP positon | P_value | Allel effect | PVE |
| --- | --- | --- | --- | --- |
| The ear leaf | | | | |
| 1 | 9643690 | 1.40E-05 | 2.127154 | 13.2% |
| 1 | 14451048 | 4.93E-05 | 1.847668 |  |
| 1 | 15018542 | 2.16E-05 | 1.807882 |  |
| 1 | 15018570 | 8.43E-06 | 1.882179 |  |
| 1 | 15018580 | 8.43E-06 | 1.882179 |  |
| 1 | 202469709 | 1.65E-05 | 2.007578 |  |
| 1 | 228881258 | 4.31E-05 | 1.82378 |  |
| 1 | 229523444 | 7.02E-07 | 2.007166 |  |
| 1 | 229523457 | 7.02E-07 | 2.007166 |  |
| 1 | 229523538 | 7.02E-07 | 2.007166 |  |
| 1 | 229523539 | 3.33E-08 | 2.297721 |  |
| 1 | 229523543 | 7.02E-07 | 2.007166 |  |
| 1 | 229523572 | 7.02E-07 | 2.007166 |  |
| 1 | 229523592 | 7.02E-07 | 2.007166 |  |
| 1 | 229523597 | 7.02E-07 | 2.007166 |  |
| 1 | 229669577 | 6.69E-05 | 1.736872 |  |
| 1 | 230669526 | 4.62E-06 | 1.865408 |  |
| 1 | 230673682 | 1.27E-06 | 1.990803 |  |
| 1 | 230673714 | 1.27E-06 | 1.990803 |  |
| 1 | 230673755 | 4.78E-07 | 2.194188 |  |
| 1 | 230717740 | 5.43E-06 | 1.883745 |  |
| 1 | 230828844 | 5.02E-07 | 2.049565 |  |
| 1 | 230850686 | 5.95E-06 | 2.244403 |  |
| 1 | 265714478 | 4.54E-05 | 1.870981 |  |
| 2 | 66227671 | 1.70E-05 | 1.637064 |  |
| 2 | 66227685 | 1.70E-05 | 1.637064 |  |
| 2 | 66227694 | 1.78E-05 | 1.636044 |  |
| 2 | 66227733 | 1.67E-05 | 1.637814 |  |
| 4 | 3376467 | 1.03E-05 | -3.388876 |  |
| 4 | 3376469 | 1.05E-05 | -3.383255 |  |
| 4 | 3376487 | 1.15E-05 | -3.377891 |  |
| 4 | 3376491 | 1.15E-05 | -3.377891 |  |
| 4 | 3376512 | 1.05E-05 | -3.383255 |  |
| 4 | 3376520 | 1.05E-05 | -3.383255 |  |
| 4 | 21070855 | 1.70E-05 | 2.465252 |  |
| 4 | 24605083 | 5.13E-05 | 1.419734 |  |
| 4 | 25013232 | 1.22E-05 | 1.625606 |  |
| 4 | 25795675 | 6.01E-05 | 1.889353 |  |
| 4 | 43801418 | 2.73E-05 | 1.856083 |  |
| 4 | 127839799 | 5.19E-06 | 2.053439 |  |
| 4 | 127839803 | 6.16E-06 | 2.038631 |  |
| 4 | 166471806 | 4.80E-06 | 2.168931 |  |
| 4 | 166596960 | 2.04E-05 | 1.677807 |  |
| 4 | 184523908 | 1.23E-05 | 1.939067 |  |
| 4 | 184671527 | 2.60E-05 | 1.902369 |  |
| 4 | 184671568 | 6.79E-05 | 1.815901 |  |
| 4 | 184671569 | 6.79E-05 | 1.815901 |  |
| 4 | 186509123 | 4.35E-05 | 1.841682 |  |
| 4 | 190073323 | 8.66E-07 | 2.060167 |  |
| 4 | 192647342 | 8.57E-07 | 1.915412 |  |
| 4 | 192647450 | 4.99E-07 | 2.179523 |  |
| 4 | 192647456 | 6.93E-06 | 1.822009 |  |
| 4 | 192658034 | 1.01E-06 | 1.880599 |  |
| 4 | 192658160 | 7.23E-07 | 1.881571 |  |
| 4 | 193802990 | 4.45E-07 | 2.348242 |  |
| 4 | 196571110 | 1.21E-06 | 2.140918 |  |
| 4 | 196778527 | 1.50E-06 | 2.297541 |  |
| 4 | 196888812 | 1.57E-07 | 2.430204 |  |
| 4 | 211051226 | 6.54E-05 | 1.679174 |  |
| 4 | 213362726 | 3.28E-06 | 2.2629 |  |
| 4 | 213362752 | 3.28E-06 | 2.2629 |  |
| 4 | 214506597 | 1.81E-05 | 2.02537 |  |
| 4 | 214976527 | 2.20E-06 | 2.121752 |  |
| 4 | 215256872 | 2.58E-05 | 1.942737 |  |
| 4 | 215478044 | 9.55E-06 | 2.09961 |  |
| 4 | 215478529 | 4.71E-06 | 2.15265 |  |
| 4 | 219726797 | 1.66E-05 | 2.065272 |  |
| 4 | 221174966 | 3.99E-05 | 2.006612 |  |
| 4 | 221710221 | 5.50E-05 | 1.945745 |  |
| 4 | 222913194 | 7.77E-06 | 2.147577 |  |
| 4 | 223005470 | 1.10E-05 | 2.089641 |  |
| 4 | 223590425 | 5.98E-05 | 1.761268 |  |
| 4 | 223649491 | 2.50E-06 | 2.039012 |  |
| 4 | 223649580 | 5.71E-05 | 1.729612 |  |
| 4 | 223673216 | 4.51E-05 | 1.784718 |  |
| 4 | 223753077 | 3.87E-06 | 2.07372 |  |
| 4 | 224642040 | 5.44E-05 | 1.769433 |  |
| 4 | 224935584 | 1.57E-05 | 2.196149 |  |
| 4 | 225121873 | 2.25E-05 | 2.014215 |  |
| 4 | 225576642 | 6.13E-05 | 1.739665 |  |
| 4 | 225937122 | 5.32E-05 | 1.887831 |  |
| 4 | 225952618 | 6.44E-05 | 1.769685 |  |
| 4 | 225952883 | 1.11E-05 | 1.849359 |  |
| 4 | 225954509 | 1.14E-05 | 1.966764 |  |
| 4 | 225954511 | 1.27E-05 | 1.957388 |  |
| 4 | 225957513 | 2.01E-05 | 1.81323 |  |
| 4 | 225957712 | 5.00E-05 | 1.754392 |  |
| 4 | 226176001 | 1.95E-05 | 2.028977 |  |
| 4 | 226607641 | 2.13E-06 | 2.20149 |  |
| 4 | 227789562 | 1.96E-05 | 2.013701 |  |
| 4 | 228933194 | 1.84E-05 | 1.973988 |  |
| 4 | 228941879 | 6.29E-06 | 2.088703 |  |
| 4 | 228993980 | 1.27E-06 | 2.114682 |  |
| 4 | 228994014 | 1.44E-06 | 2.091252 |  |
| 4 | 228994034 | 1.27E-06 | 2.114682 |  |
| 4 | 228994044 | 1.27E-06 | 2.114682 |  |
| 4 | 228994097 | 1.27E-06 | 2.114682 |  |
| 4 | 228994108 | 1.27E-06 | 2.114682 |  |
| 4 | 228994134 | 1.27E-06 | 2.114682 |  |
| 4 | 228994160 | 1.27E-06 | 2.114682 |  |
| 4 | 228994171 | 1.27E-06 | 2.114682 |  |
| 4 | 230599406 | 3.07E-05 | 1.982904 |  |
| 4 | 230813637 | 1.14E-06 | 2.078727 |  |
| 4 | 230853542 | 5.52E-06 | 2.100482 |  |
| 4 | 230853667 | 5.79E-06 | 2.110729 |  |
| 4 | 230853668 | 5.79E-06 | 2.110729 |  |
| 4 | 230988998 | 1.80E-06 | 2.156463 |  |
| 4 | 232927341 | 4.76E-06 | 2.17301 |  |
| 6 | 30235752 | 1.01E-05 | 2.10535 |  |
| 6 | 30235773 | 1.05E-05 | 2.10107 |  |
| 6 | 160596319 | 5.90E-05 | 1.901703 |  |
| 6 | 160596359 | 5.90E-05 | 1.901703 |  |
| 6 | 171164699 | 2.56E-05 | 2.009122 |  |
| 7 | 134232411 | 1.15E-05 | -2.540007 |  |
| 8 | 56540381 | 6.47E-05 | 1.66062 |  |
| 9 | 16748148 | 6.66E-05 | 1.912386 |  |
| 9 | 16867650 | 6.83E-05 | 1.92405 |  |
| 9 | 16867678 | 3.79E-05 | 2.007845 |  |
| 9 | 16963618 | 6.57E-05 | 1.909212 |  |
| 9 | 17503563 | 4.49E-05 | 1.939514 |  |
| 9 | 17503593 | 4.49E-05 | 1.939514 |  |
| 9 | 17503785 | 3.46E-05 | 1.903303 |  |
| 9 | 18610979 | 1.16E-05 | 2.049679 |  |
| 9 | 20926852 | 2.64E-06 | 2.179395 |  |
| 9 | 21278371 | 5.13E-05 | 1.92335 |  |
| 9 | 22048713 | 3.96E-06 | 2.191843 |  |
| 9 | 25165918 | 5.76E-05 | 1.756386 |  |
| 9 | 25212103 | 1.97E-05 | 2.064632 |  |
| 9 | 25235605 | 5.67E-05 | 1.776658 |  |
| 9 | 154467241 | 2.02E-05 | 1.977839 |  |
| 9 | 154467304 | 2.02E-05 | 1.977839 |  |
| 10 | 67258610 | 6.07E-05 | 1.75163 |  |
| 10 | 67258618 | 6.07E-05 | 1.75163 |  |
| The second leaf above the ear leaf | | | | |
| 4 | 19748788 | 5.21E-05 | 2.242218 | 8.6% |
| 4 | 25795675 | 7.61E-06 | 2.43611 |  |
| 4 | 26334682 | 1.86E-05 | 2.317754 |  |
| 4 | 26471662 | 6.44E-05 | 2.033089 |  |
| 4 | 92764348 | 6.53E-05 | 2.284062 |  |
| 4 | 92764575 | 5.54E-05 | 2.302426 |  |
| 4 | 150510753 | 9.34E-06 | 2.512297 |  |
| 4 | 193802990 | 3.94E-05 | 2.324899 |  |
| 4 | 196888812 | 1.93E-05 | 2.401208 |  |
| 6 | 125962083 | 3.69E-05 | -2.854578 |  |
| 6 | 171164699 | 6.08E-05 | 2.204936 |  |
| 7 | 33088106 | 4.33E-05 | 1.47352 |  |
| 7 | 64349516 | 6.37E-05 | 1.522516 |  |
| 7 | 64349610 | 2.77E-05 | 1.606243 |  |
| 7 | 96070685 | 5.78E-05 | 1.383733 |  |
| 7 | 98561673 | 5.98E-05 | 1.289922 |  |
| 7 | 108660424 | 2.53E-05 | 1.324042 |  |
| 7 | 108660453 | 4.24E-05 | 1.291738 |  |
| 7 | 108759792 | 5.31E-05 | 1.266576 |  |
| 7 | 134232411 | 6.96E-05 | -2.689883 |  |
| 9 | 20926852 | 6.34E-05 | 2.194496 |  |
| 9 | 128056619 | 6.46E-05 | 1.318598 |  |
| 9 | 156484518 | 3.05E-05 | 0.9678893 |  |

**Table S2: Genes screened for Significant SNPs of the ear leaf and the second leaf above the ear leaf.**

| Chromosome | start | end | Gene ID | Description |
| --- | --- | --- | --- | --- |
| The ear leaf | | | | |
| 1 | 14448014 | 14448412 | Zm00001d027819 | embryo surrounding region2 |
| 1 | 15012548 | 15013896 | Zm00001d027844 | Ribosomal L18p/L5e family protein |
| 1 | 15013950 | 15018699 | Zm00001d027845 | Pentatricopeptide repeat-containing protein mitochondrial |
| 1 | 202456321 | 202462069 | Zm00001d031807 |  |
| 1 | 202464156 | 202467819 | Zm00001d031808 | NAD(P)-binding Rossmann-fold superfamily protein |
| 1 | 202468442 | 202472613 | Zm00001d031809 | Metal-independent phosphoserine phosphatase |
| 1 | 229515984 | 229517919 | Zm00001d032527 | Hydroxycinnamoyl transferase13 |
| 1 | 230664256 | 230666245 | Zm00001d032565 | Microtubule-associated protein RP/EB family member 1C |
| 1 | 230666267 | 230672973 | Zm00001d032566 |  |
| 1 | 230714286 | 230732552 | Zm00001d032567 | Mediator of RNA polymerase II transcription subunit 16 |
| 1 | 230852598 | 230853237 | Zm00001d032569 | Ras-related protein RABA6a |
| 1 | 230853577 | 230855383 | Zm00001d032570 | soluble epoxide hydrolase |
| 1 | 9637254 | 9638102 | Zm00001d027636 | Dirigent protein 16 |
| 1 | 9638241 | 9644168 | Zm00001d027637 |  |
| 4 | 166465152 | 166469789 | Zm00001d051677 | KH domain-containing protein |
| 4 | 166470978 | 166481012 | Zm00001d051678 | WD40 G-beta domain containing family protein |
| 4 | 166593637 | 166595477 | Zm00001d051680 |  |
| 4 | 166604240 | 166605598 | Zm00001d051681 | Glycosyltransferase 6 |
| 4 | 166606260 | 166610196 | Zm00001d051682 | DNA-directed RNA polymerases IV and V subunit 4 |
| 4 | 184666747 | 184669386 | Zm00001d052247 | Shikimate kinase 1 chloroplastic |
| 4 | 186500641 | 186503477 | Zm00001d052303 | D-aminoacid aminotransferase-like PLP-dependent enzymes superfamily protein |
| 4 | 190062610 | 190072524 | Zm00001d052436 | ubiquinol-cytochrome C reductase UQCRX/QCR9-like family protein |
| 4 | 190065405 | 190067801 | Zm00001d052437 | L-aminoadipate-semialdehyde dehydrogenase-phosphopantetheinyl transferase |
| 4 | 190068552 | 190071190 | Zm00001d052438 | Putative calcium-dependent protein kinase family protein |
| 4 | 190073152 | 190083229 | Zm00001d052439 | RNA binding (RRM/RBD/RNP motifs) family protein |
| 4 | 192655475 | 192657030 | Zm00001d052542 | Dual specificity protein phosphatase Diacylglycerol kinase catalytic region |
| 4 | 192657367 | 192664592 | Zm00001d052543 | bZIP transcription factor family protein |
| 4 | 196893203 | 196896534 | Zm00001d052673 | Chalcone synthase C2 |
| 4 | 21064147 | 21064729 | Zm00001d049214 | Hevein-like preproprotein |
| 4 | 211042468 | 211043881 | Zm00001d053054 | Enoyl-CoA hydratase 2 peroxisomal |
| 4 | 211054808 | 211055547 | Zm00001d053055 | Dirigent protein 23 |
| 4 | 215474432 | 215476369 | Zm00001d053135 | D-mannose binding lectin family protein |
| 4 | 219732142 | 219732638 | Zm00001d053205 |  |
| 4 | 222969266 | 223050007 | Zm00001d053262 | calcium-dependent lipid-binding family protein |
| 4 | 223581945 | 223588791 | Zm00001d053273 | Adhesin FhaB |
| 4 | 224638609 | 224640973 | Zm00001d053294 |  |
| 4 | 224942731 | 224952442 | Zm00001d053300 |  |
| 4 | 228946356 | 228947500 | Zm00001d053389 | Glutamate--tRNA ligase chloroplastic/mitochondrial |
| 4 | 230807016 | 230807411 | Zm00001d053415 |  |
| 4 | 230820849 | 230823728 | Zm00001d053416 | Probable inactive dual specificity protein phosphatase-like |
| 4 | 232902680 | 232925350 | Zm00001d053521 |  |
| 4 | 24613816 | 24614515 | Zm00001d049290 | Beta-13-N-Acetylglucosaminyltransferase family protein |
| 6 | 160602563 | 160603687 | Zm00001d038591 | 60S ribosomal protein L10a-1 |
| 6 | 171163852 | 171169201 | Zm00001d039154 | 26S proteasome regulatory subunit RPN13 |
| 9 | 154459210 | 154461209 | Zm00001d048317 |  |
| 9 | 154463094 | 154468191 | Zm00001d048318 | Protoheme IX farnesyltransferase |
| 9 | 154468684 | 154472507 | Zm00001d048319 | Probable mannan synthase 7 |
| 9 | 154473757 | 154480081 | Zm00001d048320 | Pentatricopeptide repeat-containing protein mitochondrial |
| 9 | 16742278 | 16746086 | Zm00001d045232 | Carotenoid 910(9'10')-cleavage dioxygenase 1 |
| 9 | 16743641 | 16749848 | Zm00001d045233 | 2-oxoglutarate (2OG) and Fe(II)-dependent oxygenase superfamily protein |
| 9 | 16750880 | 16752190 | Zm00001d045235 |  |
| 9 | 17495380 | 17496111 | Zm00001d045268 | Histone H3.2 |
| 9 | 17510977 | 17523858 | Zm00001d045269 | Multidrug resistance-associated protein3 |
| 9 | 20934388 | 20935797 | Zm00001d045408 | S-adenosyl-L-methionine-dependent methyltransferases superfamily protein |
| 9 | 25240624 | 25249861 | Zm00001d045516 | Probable transcriptional regulator SLK2 |
| The second leaf above the ear leaf | | | | |
| 4 | 150498265 | 150503275 | Zm00001d051249 | Elicitor-responsive protein 3 |
| 4 | 196893203 | 196896534 | Zm00001d052673 | Chalcone synthase C2 |
| 4 | 19755800 | 19757563 | Zm00001d049187 | phosphogluconate dehydrogenase3 |
| 4 | 26332755 | 26337307 | Zm00001d049324 | Nucleobase-ascorbate transporter 2 |
| 4 | 26475602 | 26481894 | Zm00001d049326 | Probable galacturonosyltransferase 4 |
| 6 | 171163852 | 171169201 | Zm00001d039154 | 26S proteasome regulatory subunit RPN13 |
| 7 | 108764029 | 108774978 | Zm00001d020355 | NPK1-related protein kinase 2 |
| 7 | 33080186 | 33084808 | Zm00001d019424 | Replication protein-like |
| 7 | 98568267 | 98580808 | Zm00001d020189 | hAT transposon superfamily protein |
| 9 | 128051621 | 128058240 | Zm00001d047373 | Ubiquitin domain-containing protein DSK2b |
| 9 | 128061736 | 128062497 | Zm00001d047374 | Cyclin-dependent kinase B1-1 |
| 9 | 156475398 | 156476045 | Zm00001d048441 | Protein PAM68 chloroplastic |
| 9 | 156483137 | 156492346 | Zm00001d048442 | Ubiquitin carboxyl-terminal hydrolase-related protein |
| 9 | 20934388 | 20935797 | Zm00001d045408 | S-adenosyl-L-methionine-dependent methyltransferases superfamily protein |

**Table S3. Genes scanned with Significant SNPs of the ear leaf and the second leaf above the ear leaf.**

| Chromesome | SNP positon | P_value | Associated Genes |
| --- | --- | --- | --- |
| The ear leaf | | | |
| 1 | 9643690 | 1.40E-05 | Zm00001d027636, Zm00001d027637 |
| 1 | 14451048 | 4.93E-05 | Zm00001d027819 |
| 1 | 15018542 | 2.16E-05 | Zm00001d027844, Zm00001d027845 |
| 1 | 15018570 | 8.43E-06 | Zm00001d027844, Zm00001d027845 |
| 1 | 15018580 | 8.43E-06 | Zm00001d027844, Zm00001d027845 |
| 1 | 202469709 | 1.65E-05 | Zm00001d031807, Zm00001d031808, Zm00001d031809 |
| 1 | 229523444 | 7.02E-07 | Zm00001d032527 |
| 1 | 229523457 | 7.02E-07 | Zm00001d032527 |
| 1 | 229523538 | 7.02E-07 | Zm00001d032527 |
| 1 | 229523539 | 3.33E-08 | Zm00001d032527 |
| 1 | 229523543 | 7.02E-07 | Zm00001d032527 |
| 1 | 229523572 | 7.02E-07 | Zm00001d032527 |
| 1 | 229523592 | 7.02E-07 | Zm00001d032527 |
| 1 | 229523597 | 7.02E-07 | Zm00001d032527 |
| 1 | 230669526 | 4.62E-06 | Zm00001d032565, Zm00001d032566 |
| 1 | 230673682 | 1.27E-06 | Zm00001d032565, Zm00001d032566 |
| 1 | 230673714 | 1.27E-06 | Zm00001d032565, Zm00001d032566 |
| 1 | 230673755 | 4.78E-07 | Zm00001d032565, Zm00001d032566 |
| 1 | 230717740 | 5.43E-06 | Zm00001d032567 |
| 1 | 230850686 | 5.95E-06 | Zm00001d032569, Zm00001d032570 |
| 4 | 21070855 | 1.70E-05 | Zm00001d049214 |
| 4 | 24605083 | 5.13E-05 | Zm00001d049290 |
| 4 | 166471806 | 4.80E-06 | Zm00001d051677, Zm00001d051678 |
| 4 | 166596960 | 2.04E-05 | Zm00001d051680, Zm00001d051681, Zm00001d051682 |
| 4 | 184671527 | 2.60E-05 | Zm00001d052247 |
| 4 | 184671568 | 6.79E-05 | Zm00001d052247 |
| 4 | 184671569 | 6.79E-05 | Zm00001d052247 |
| 4 | 186509123 | 4.35E-05 | Zm00001d052303 |
| 4 | 190073323 | 8.66E-07 | Zm00001d052436, Zm00001d052437, Zm00001d052438, Zm00001d052439 |
| 4 | 192658034 | 1.01E-06 | Zm00001d052542,Zm00001d052543 |
| 4 | 192658160 | 7.23E-07 | Zm00001d052542,Zm00001d052543 |
| 4 | 196888812 | 1.57E-07 | Zm00001d052673 |
| 6 | 160596319 | 5.90E-05 | Zm00001d038591 |
| 6 | 160596359 | 5.90E-05 | Zm00001d038591 |
| 6 | 171164699 | 2.56E-05 | Zm00001d039154 |
| 9 | 16748148 | 6.66E-05 | Zm00001d045232, Zm00001d045233, Zm00001d045235 |
| 9 | 17503563 | 4.49E-05 | Zm00001d045268, Zm00001d045269 |
| 9 | 17503593 | 4.49E-05 | Zm00001d045268, Zm00001d045269 |
| 9 | 17503785 | 3.46E-05 | Zm00001d045268, Zm00001d045269 |
| 9 | 20926852 | 2.64E-06 | Zm00001d045408 |
| 9 | 25235605 | 5.67E-05 | Zm00001d045516 |
| 9 | 154467241 | 2.02E-05 | Zm00001d048318, Zm00001d048319 |
| 9 | 154467304 | 2.02E-05 | Zm00001d048318, Zm00001d048319 |
| The second leaf above the ear leaf | | | |
| 4 | 19748788 | 5.21E-05 | Zm00001d049187 |
| 4 | 26334682 | 1.86E-05 | Zm00001d049324 |
| 4 | 26471662 | 6.44E-05 | Zm00001d049324 |
| 4 | 150510753 | 9.34E-06 | Zm00001d051249 |
| 4 | 196888812 | 1.93E-05 | Zm00001d052673 |
| 6 | 171164699 | 6.08E-05 | Zm00001d039154 |
| 7 | 33088106 | 4.33E-05 | Zm00001d019424 |
| 7 | 108759792 | 5.31E-05 | Zm00001d020355 |
| 9 | 20926852 | 6.34E-05 | Zm00001d045408 |
| 9 | 128056619 | 6.46E-05 | Zm00001d047373, Zm00001d047374 |
| 9 | 156484518 | 3.05E-05 | Zm00001d048441, Zm00001d048442 |

**Table S4. Number of most important SNP locus types in the three populations.**

|  | Alleles type | |
| --- | --- | --- |
| The ear leaf | TT | GG |
| RIL-YML32 | 150 | 0 |
| RIL-YML226 | 94 | 21 |
| RIL-D39 | 179 | 0 |
| The second leaf above the ear |  | |
| RIL-YML32 | 149 | 0 |
| RIL-YML226 | 94 | 21 |
| RIL-D39 | 179 | 0 |

**Table S5. GO enrichment result of candidate genes.**

| Chromosome | Gene ID | GO accession | GO term |
| --- | --- | --- | --- |
| The ear leaf | | | |
| 1 | Zm00001d027819 | GO:0005515 | protein binding |
| 1 | Zm00001d027844 | GO:0043228 | non-membrane-bounded organelle |
| 1 | Zm00001d027845 |  |  |
| 1 | Zm00001d031807 |  |  |
| 1 | Zm00001d031808 |  |  |
| 1 | Zm00001d031809 | GO:0016787 | hydrolase activity |
| 1 | Zm00001d032527 | GO:0003824 | catalytic activity |
| 1 | Zm00001d032565 | GO:0043232 | intracellular non-membrane-bounded organelle |
| 1 | Zm00001d032566 |  |  |
| 1 | Zm00001d032567 | GO:0050896 | response to stimulus |
| 1 | Zm00001d032569 | GO:0016787 | hydrolase activity |
| 1 | Zm00001d032570 | GO:0016787 | hydrolase activity |
| 1 | Zm00001d027636 | GO:0003824 | catalytic activity |
| 1 | Zm00001d027637 |  |  |
| 4 | Zm00001d051677 | GO:1901564 | organonitrogen compound metabolic process |
| 4 | Zm00001d051678 | GO:0043228 | non-membrane-bounded organelle |
| 4 | Zm00001d051680 | GO:0003824 | catalytic activity |
| 4 | Zm00001d051681 | GO:0043227 | membrane-bounded organelle |
| 4 | Zm00001d051682 | GO:0003824 | catalytic activity |
| 4 | Zm00001d052247 | GO:0043227 | membrane-bounded organelle |
| 4 | Zm00001d052303 | GO:0003824 | catalytic activity |
| 4 | Zm00001d052436 | GO:0043229 | intracellular organelle |
| 4 | Zm00001d052437 | GO:1901576 | organic substance biosynthetic process |
| 4 | Zm00001d052438 | GO:0016740 | transferase activity |
| 4 | Zm00001d052439 | GO:0044446 | intracellular organelle part |
| 4 | Zm00001d052542 | GO:0016301 | kinase activity |
| 4 | Zm00001d052543 | GO:0010556 | regulation of macromolecule biosynthetic process |
| 4 | Zm00001d052673 | GO:1901576 | organic substance biosynthetic process |
| 4 | Zm00001d049214 | GO:0050896 | response to stimulus |
| 4 | Zm00001d053054 |  |  |
| 4 | Zm00001d053055 | GO:0003824 | catalytic activity |
| 4 | Zm00001d053135 | GO:0003824 | catalytic activity |
| 4 | Zm00001d053205 | GO:0016070 | RNA metabolic process |
| 4 | Zm00001d053262 | GO:0044699 | single-organism process |
| 4 | Zm00001d053273 |  |  |
| 4 | Zm00001d053294 |  |  |
| 4 | Zm00001d053300 | GO:2000112 | regulation of cellular macromolecule biosynthetic process |
| 4 | Zm00001d053389 | GO:1901360 | organic cyclic compound metabolic process |
| 4 | Zm00001d053415 |  |  |
| 4 | Zm00001d053416 |  |  |
| 4 | Zm00001d053521 | GO:0097159 | organic cyclic compound binding |
| 4 | Zm00001d049290 | GO:0016740 | transferase activity |
| 6 | Zm00001d038591 | GO:1901566 | organonitrogen compound biosynthetic process |
| 6 | Zm00001d039154 | GO:0043229 | intracellular organelle |
| 9 | Zm00001d048317 |  |  |
| 9 | Zm00001d048318 | GO:0043232 | intracellular non-membrane-bounded organelle |
| 9 | Zm00001d048319 | GO:0016021 | integral component of membrane |
| 9 | Zm00001d048320 |  |  |
| 9 | Zm00001d045232 | GO:0044446 | intracellular organelle part |
| 9 | Zm00001d045233 | GO:0003824 | catalytic activity |
| 9 | Zm00001d045235 |  |  |
| 9 | Zm00001d045268 | GO:0043229 | intracellular organelle |
| 9 | Zm00001d045269 | GO:0043229 | intracellular organelle |
| 9 | Zm00001d045408 | GO:0031224 | intrinsic component of membrane |
| 9 | Zm00001d045516 | GO:0032991 | macromolecular complex |
| The second leaf above the ear leaf | | | |
| 4 | Zm00001d051249 |  |  |
| 4 | Zm00001d052673 | GO:0003824 | catalytic activity |
| 4 | Zm00001d049187 | GO:1901564 | organonitrogen compound metabolic process |
| 4 | Zm00001d049324 | GO:0016021 | integral component of membrane |
| 4 | Zm00001d049326 | GO:0044446 | intracellular organelle part |
| 6 | Zm00001d039154 | GO:0048518 | positive regulation of biological process |
| 7 | Zm00001d020355 | GO:0044710 | single-organism metabolic process |
| 7 | Zm00001d019424 |  |  |
| 7 | Zm00001d020189 | GO:0005515 | protein binding |
| 9 | Zm00001d047373 | GO:0005515 | protein binding |
| 9 | Zm00001d047374 | GO:0032991 | macromolecular complex |
| 9 | Zm00001d048441 | GO:0043229 | intracellular organelle |
| 9 | Zm00001d048442 | GO:0071704 | organic substance metabolic process |
| 9 | Zm00001d045408 | GO:0031224 | intrinsic component of membrane |
